# Physical activity and screen time in outside school hours care services across Australia: current versus best practice

**DOI:** 10.1186/s12889-022-13135-7

**Published:** 2022-04-07

**Authors:** Rosa Virgara, Anna Phillips, Lucy K. Lewis, Mandy Richardson, Carol A. Maher

**Affiliations:** 1 Allied Health and Human Performance, University of South Australia, Adelaide, SA Australia; 2 Caring Futures Institute, College of Nursing and Health Sciences, Flinders University, Bedford Park, SA Australia; 3 Department for Education, Government of South Australia, Adelaide, SA Australia; 4 University of South Australia – City East Campus, GPO Box 2471, Adelaide, SA 5001 Australia

**Keywords:** Physical activity, Screen time, Outside school hours care, Best practice, Guidelines

## Abstract

**Background:**

Outside school hours care (OSHC) is accessed by millions of children internationally. Recently, physical activity and screen time guidelines in OSHC were developed. This study described the current physical activity and screen time scheduling in Australian OSHC, obtained sector feedback on the guidelines and compared current- with best-practice.

**Methods:**

A cross-sectional online survey was administered to *n* = 3551 Australian OSHC directors. Participants reported scheduling for physical activity and screen time opportunities in before- and after-school care. Feedback was sought on the new guidelines, including barriers and enablers for implementation. Scheduling data were used to evaluate whether services were currently meeting the new guidelines; that is if time allocated matched with time recommended.

**Results:**

Five hundred and sixty-six directors participated (response rate 16%). Physical activity and screen time practices varied widely (e.g., after-school physical activity opportunity ranged from 15 to 150 min, mean 74, SD 28; after-school screen time opportunity ranged from 15 to 195 min, mean 89, SD 43), with state (*p* = 0.002) and socioeconomic (based on postcode; *p* < 0.001) differences. Most participants (54–81%) agreed that the guideline’s recommended physical activity and screen time durations were appropriate, however, only 40% of participants’ OSHC services’ programs actually met the guidelines.

**Conclusions:**

Physical activity and screen time scheduling in OSHC is highly variable. Despite support for the guidelines, current scheduling practice in the majority of OSHC services surveyed do not meet best practice guidelines.

**Supplementary Information:**

The online version contains supplementary material available at 10.1186/s12889-022-13135-7.

## Background

Engaging in adequate amounts of physical activity (PA) is associated with improved cardiovascular and bone health, academic performance, and quality of life [1, 2]. Furthermore, excessive recreational screen time (ST) is associated with obesity, behavioural issues, anxiety, depression and poorer academic performance [3]. Less than half of Australian children meet the World Health Organization’s (WHO) recommendations for PA and less than a third meet the ST recommendations [4].

The outside school hours period (before and after school and the weekends) provides an opportunity for the accumulation of PA and sedentary behaviours [5, 6]. Many school-aged children around the world attend formal before- or after-school childcare, for example, 18% of children in the United States [7] and 10% in Australia [8]. The terminology used to describe before and after school care varies around the world and within countries. For example, in the UK in can be called “Childcare Outside of School Hours”, “Homework Club”, “Breakfast Club”, “After School Club” [9] or Extended Schools Access [10]; and “After School Program” or “Out-of-School Time” in the US [11]; and typically “Outside School Hours Care (OSHC)” in Australia. The length and structure of programs also varies. In Australia, before school hours care typically runs from 0630 – 0830am and after school care from 1500–1800, with flexibility in terms of how long children attend (i.e., parents can drop off and collect their child at any time during the care session). The OSHC setting holds population health potential for positively impacting children’s activity patterns. However, PA and ST practices in this setting are highly variable. For example, a 2016 Australian study found that approximately 61% of after-school care sessions were spent sedentary (range 31%—79%) and only 18% of session time was spent in MVPA (range 4%—49%) [12]. OSHC directors stated they wanted clear policy and guidance to help plan PA and ST programming in OSHC [12]. Similar results have been noted in the US. A 2015 review of practice in 19 after school care programs reporting " compliant (75% to 98% meet LVPA activity policies) or largely fail to provide sufficient levels of activity (0.3% to 27% meet MVPA policies)”p.6. [7].

Guidelines are evidence-based recommendations to encourage best practice [13]. A recent international scoping review identified nine PA and ST guidelines designed for the OSHC setting [14]. These guidelines varied in terms of the durations and intensities of PA and ST recommended; were published in grey-literature; and none followed rigorous guideline development methods [14]. Over 2019–2020, our team worked with stakeholders to develop the first PA and ST guidelines for Australian OSHC, following the Grading of Recommendations Assessment, Development and Evaluation (GRADE) guideline development methodology [15], and informed by a scoping [14] and Cochrane review of PA interventions in OSHC [16] (supplementary file 1).

Guideline implementation can be bolstered when current practice, and how this meets best-practice guidelines, is understood (i.e. defining the evidence-practice gap); when practitioners have had the opportunity to comment or feedback on the guidelines (improving credibility); and understanding the barriers and enablers to implementation [17]. Accordingly, this study aimed to (i) describe current practice regarding scheduling of PA and ST during before and after-school care in Australian OSHC services; (ii) present the newly-developed OSHC-sector PA and ST guidelines [18] to OSHC directors to seek their feedback; (iii) explore enablers and barriers to guideline implementation; and (iv) determine whether OSHC services were currently meeting the guideline recommendations for PA and ST.

## Methods

A cross-sectional online survey was conducted from June to August 2020. Ethical approval was provided by the University’s institution ethics board (no. 202898).

A 150-item purpose-designed survey instrument was developed (supplementary file 2) by the research team which collected:

Location (state), average daily attendance, and the care sessions provided (before-school, after-school). Socioeconomic status was determined from postcode, based upon Australian Bureau of Statistics’ Socio-Economic Indexes for Areas (SEIFA) [19]. It took approximately 30 min to complete.

Activities offered and typical duration of the activities were captured using a items developed in a previous OSHC study [12] Participants were invited to report the availability of various activities in three domains: general (9 items – e.g. Lego, role play), screen-based activities (7 items—e.g. TV/DVD viewing) and physical activities (7 items – e.g. outdoor play, sports equipment). For each activity, participants were asked if the activity was offered daily, if so, they then reported the time it was offered in 15-min increments. The survey items were intentionally ordered to capture general activities first, and then ST and PA, to reduce the emphasis on PA and ST, to minimise social desirability bias [20]. The scheduling tool has been validated relative to directly observed PA (*r* = 0.41) and ST (*r* = 0.73) [21].

The OSHC-sector PA and ST guidelines (supplementary file 1) and elaboration document [18] were presented to participants, and participants’ perceptions were explored through 16 items. The first question was open-ended and invited overall feedback on the guidelines and elaboration document. Likert items were used to obtain feedback on the durations of PA and ST recommendations for before-school care, after-school care, and vacation care, with the response options “too high”, "just right" or "too low". Additional comments regarding the duration and wording of the PA and ST recommendations were captured using open-ended items. Two items explored OSHC directors' confidence to implement the PA and ST guidelines using a 5-point Likert scale (1: not at all confident – 5: extremely confident).

The importance of potential barriers (e.g. behaviour management issues, workplace culture, children's attitudes) and enablers (e.g. staff training, workplace policy and staff knowledge) to guideline uptake were ranked (using based on factors identified from the Delphi study [18]). Open-ended items allowed participants to suggest additional barriers and enablers.

All OSHC directors in any metropolitan, rural, or remote location in both public and private settings were eligible. A list of email addresses for all Australian OSHC service was obtained from the Australian Children’s Education and Care Quality Authority (ACECQA) website. The survey was open for 4 weeks, with weekly email invitation reminders. In addition, the survey was distributed to members of National Outside School Hours Services Alliance (NOSHSA)—the peak Australian OSHC body, and it was posted on OSHC specific Facebook® groups (OSHC/OOSH Network, OSHC Educators, OOSH Connect and Community Childcare Association). A $100 random prize draw was offered for survey participation.

All responses were downloaded into SPSS [22]. Demographic and scheduling data were analysed descriptively. Non-responder bias was examined by comparing responders' and non-responders' SEIFA and state using chi-square.

To calculate the total amount of time scheduled for PA and ST in each OSHC service during before and/or after-school care sessions, the total number of 15-min timeslots offering any PA or recreational ST, respectively, were summed.

ANOVA was used to determine whether the amount of time scheduled for PA varied by SES and state (normal distribution), whilst Kruskal–Wallis was used to examine whether ST practices varied by SES or state (because data were skewed data). In each case, two models were run: one for before-school and one for after-school care.

The PA and ST duration data were then used to calculate whether each OSHC service met each component of the guidelines, i.e., before-school PA scheduled opportunity ≥ 45 min; before-school ST scheduled opportunity ≤ 30 min; after-school PA scheduled opportunity ≥ 90 min; after-school ST scheduled opportunity ≤ 60 min. From this, the percentage of OSHC services meeting all four guideline components was calculated. ANOVA with Tukey Post Hoc testing was used to determine whether the number of guidelines components met varied according to state and SES tertile. For all statistical analyses, significance was set at *p* < 0.05.

Free text responses to open-ended questions were compiled and categorised into naturally emerging themes by the first author (RV) and cross-checked by the senior author (CM).

## Results

Three thousand five hundred fifty one survey invitations were sent to unique email addresses listed for before and/or after-school care providers on the ACECQA database. After the first email, one major OSHC provider asked for all its services to be removed from the survey distribution list, leaving *n* = 3518. A total of 566 OSHC directors (representing 566 discrete OSHC services) participated (response rate of 16%).

All Australian states and territories were represented, with most respondents from New South Wales (29%), South Australia (23%) and Queensland (19%). Results are summarised in Table 1.

**Table 1:** Demographic characteristics

Number of participants	Daily attendance mean (SD)	Provides before-school care (n)	Provides after-school care (n)	SES (SEIFA mean, SD)
Australia *n* = 566	56 (48)	491	477	1009 (65)
Australian Capital Territory (*n* = 10)	84 (45)	7	9	1073 (18)
New South Wales (*n* = 164)	66 (51)	139	143	1020 (67)
Northern Territory (*n* = 7)	80 (62)	5	7	981 (11)
Queensland (*n* = 109)	69 (60)	100	92	994 (65)
South Australia (*n* = 128)	43 (30)	120	113	999 (69)
Tasmania (*n* = 10)	38 (21)	9	7	983 (43)
Victoria (*n* = 96)	45 (45)	75	72	1012 (60)
Western Australia (*n* = 42)	36 (25)	36	34	1021 (43)

Responders and non-responders differed based on state (*X*^2^(7, *n* = 3425) = 102.6, *p* < 0.0001), with services based in South Australia more likely to respond compared to other states. However, they did not differ based on socioeconomic status (*X*^2^(2, *n* = 3425) = 0.149, *p* = 0.93).

The time scheduled for PA ranged widely in both before- and after-school, from 0 to 150 min (mean 63, SD 37) and 0 to 210 min (mean 127, SD 39) respectively (Table 2). Of note, 14% (*n* = 60) of services did not schedule any PA during before-school care. A small number (*n* = 2, < 1%) did not schedule PA in after-school care. Amongst services reporting any scheduled time for PA, it ranged from 30 to 210 min (mean 128, SD 38). The amount of time scheduled for PA (Table 2) differed significantly by state (before-school care F = 3.903, p = 0.000; after-school care F = 3.311, *p* = 0.002), but not SES (before-school care F = 1.581, *p* = 0.207; after-school care F = 1.980, *p* = 0.150). The most common types of PA offered during before- and after-school care were outdoor free play (e.g., on sports fields, using sports equipment and outdoor playground play).

**Table 2:** Physical activity and screen time scheduling by state

	**Physical Activity Scheduling** **(minutes per session)** **Mean (SD), [Range]**		**Screen Time Scheduling** **(minutes per session)** **Mean (SD), [Range]**	
	Before-school Care	After-school Care	Before-school Care	After-school Care
**Australia (*n* = 566)**	64 (37) [0–150]	127 (39) [0–210]	25 (39) [0–135]	51 (54) [0–195]
**Australian Capital Territory (*n* = 10)**	53 (30) [0–90]	154 (35) [90–195]	0	4 (11) [0–30]^l^
**New South Wales (*n* = 164)**	61 (39) [0–135]^a^	130 (42) [0–210]	20 (36) [0–120]^i,j^	42 (50) [0–180]^n^
**Northern Territory (*n* = 7)**	36 (25) [0–60]	122 (41) [45–165]	6 (13) [0–30]	60 (49) [0–120]
**Queensland (*n* = 109)**	81 (36) [0–150]^a, b, c,d^	127 (33) [30–195]^e^	21 (37) [0–120]	41 (53) [0–195]^m^
**South Australia (*n* = 128)**	58 (33) [0–120] ^b^	122 (39) [0–180]^f^	41 (43) [0–120]^h,i,j^	79 (55) [0–195]^k,l,m,n^
**Tasmania (*n* = 10)**	64 (36) [0–120]	178 (15) [150–195]^e,f,g^	9 (27) [0–75]	0^ k^
**Victoria (*n* = 96)**	62 (33) [0–135]^c^	117 (37) [45–195]^g^	18 (34) [0–135]^h^	40 (47) [0–150]
**Western Australia (*n* = 42)**	57 (34) [0–105]^d^	134 (38) [45–195]	31 (41) [0–105]	64 (64) [0–180]

^a^^−^^g^ shared letters denote statistically significant difference from one another when assessed with Tukey Post Hoc testing *p* < 0.05, ^k−n^ shared letters denote statistically significant difference from one another when assessed with Kruskall-Wallis Pairwise post hoc testing *p* < 0.05

The time scheduled for screen-based activities ranged widely, from 0 to 135 min in before-school care (mean 25, SD 39), and 0 to 195 min (mean 51, SD 54) in after-school care (Table 2). Sixty percent of services did not offer ST during before-school, and 30% did not offer it after-school. Amongst services reporting scheduling ST, it ranged from 15 to 135 min in before-school care (mean 72, SD 30) and 15 to 195 min in after-school care (mean 89, SD 43). The amount of time scheduled for screen activities differed both by state (before-school care H = 30.6, *p* = 0.000; after-school care H = 48.8, *p* = 0.000) and SES (before-school care H = 24.5, *p* = 0.000; after-school care H = 6.694, *p* = 0.035). The most common screen-based activity offered in before-school and after-school was TV/DVD viewing. Full post hoc results for PA and ST scheduling are shown in supplementary file 3.

OSHC directors rated the quantity of PA and ST recommended in the guidelines. For all six PA and ST guideline recommendations, the most common rating was "just right" (ranging from 54%—81% agreement) (supplementary file 4). OSHC directors who disagreed with the PA guideline amount rated the guideline as being too high (4 – 40% of participants) (see supplementary file 4). Likewise, OSHC directors who disagreed with the ST guideline amount again rated the guideline as too high, i.e., less ST should be offered (27–42% of participants).

Almost all (94%) of participants’ free text responses affirmed the importance and need for guidelines. The next most common feedback (92%) restated the notion that the ST guidelines should be stricter. For example, participants suggested that instead of offering ST daily, it should only be offered only for wet weather, Friday afternoons, or special occasions.

Participants ranked barriers from most important to least important: (1) lack of indoor space for active play, (2) children's attitudes, (3) behaviour management, (4) family attitudes, (5) staffing beliefs and (6) workplace culture. The enablers were ranked (from most important to least important): (1) staff understanding, (2) adequate and appropriate staff training, (3) OSHC family education and understanding of the PA and ST guidelines, and (4) workplace policy.

Overall, more than three-quarters (77%) of OSHC directors reported feeling confident (scores 4 and 5) of their ability to adjust their OSHC services programming to meet the PA time recommendations in the guidelines. Of the directors who were not confident to adjust programming (scores 1–3), only *n* = 13 provided elaborating comments, fell into two types: either than they were *already* achieving the guideline (and thus did not need to change to meet guidelines) or did not want to change their programming.

Similarly, 78% of OSHC directors reported they felt confident (scores 4 and 5 respectively) to adjust their OSHC programming to meet the ST guidelines. Again, of the directors who reported not feeling confident, their free-text comments (*n* = 11) suggested they were either already meeting the ST guidelines, or that they were not interested in changing their ST practices.

There were no statistically significant difference by state or by SES for meeting the PA guidelines in before or after-school care. However, there were significant differences by state (F = 3.284, *p* = 0.002) and SES (F = 9.206, *p* = 0.000) for services meeting before-school care ST guidelines. There were also differences by state, but not SES for meeting after-school care ST guidelines (F = 5.301, *p* = 0.000) with full post-hoc tests provided in supplementary file 5.

**Fig. 1:**
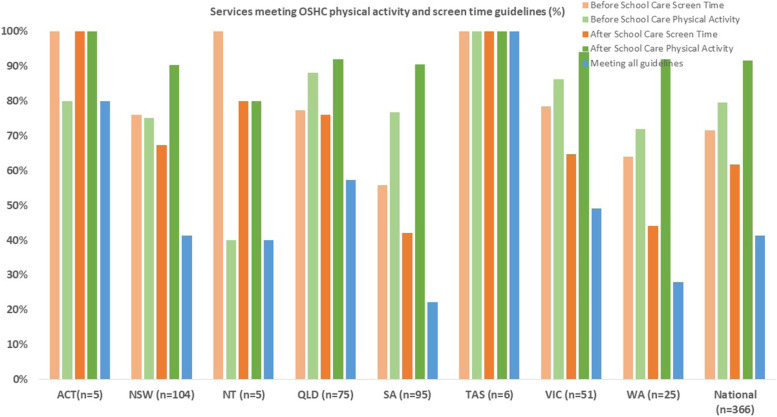
Percentage of services meeting physical activity and screen time guidelines

Compliance with each guideline component, and all four guideline components in combination, for Australia overall and by state is shown in Fig. 1. Australia-wide, 41% of OSHC services met all four guideline components, and compliance significantly differed according to state (F = 6.067, *p* = 0.000). OSHC services in Tasmania (100%) and Australian Capital Territory (100%) had highest rates of meeting all components of the guidelines, while South Australia (22%) and Western Australia (28%) had lowest rates of meeting the guidelines. There were statistically significant differences between low SES and high SES services for meeting all four guidelines (F = 8.888, *p* = 0.000). Full post-hoc results are provided in supplementary file 5.

## Discussion

In general, OSHC directors were supportive of the need for PA and ST guidelines in OSHC and the durations recommended in the draft guidelines. At present, the amount of time scheduled for PA and ST varies widely between OSHC services, with state-level and socioeconomic differences. Approximately two in five OSHC services surveyed met the new OSHC sector guidelines.

The most scheduled form of PA in OSHC throughout Australia was outdoor free play. This finding is consistent with the Curriculum Framework for School Age Care in Australia, which has a focus on the agency of children to engage in free play. The same finding was identified in earlier research conducted in South Australian OSHC services [12], and is consistent with results from our recent Delphi study [18], in which participants emphasised the importance of free play (as opposed to organised games) in OSHC. Survey results suggested that most OSHC services are offering ample children PA opportunity, underscoring the feasibility of the PA guideline recommendations.

Screen time practices in OSHC varied widely. Whilst 30–60% of OSHC services don’t offer recreational ST daily, some OSHC services offer ST for the entirety of their morning and afternoon care sessions, amounting to over five hours per day. The most common form of ST offered during OSHC was TV/DVD viewing – a finding consistent with Arundell’s 2016 systematic review, which similarly reported that TV viewing as the most common screen-based activity in after-school care [23]. There were statistically significant differences in ST scheduling by state and SES. In particular, the that OSHC services in lower SES areas had higher screen use is consistent with the general trend in previous research which consistent finds higher ST use in low SES children [24].

OSHC directors rated the leading barriers as lack of indoor space for active play, children’s attitudes, and behaviour management. This differed slightly from our recent Delphi study which suggested that educator beliefs and workplace culture would be the main barriers [18]. This difference may have been due to the range of stakeholders’ opinions, as many different end-users were involved in the Delphi study [18] (e.g. government officials, researchers, academics, parents). Further to this, there has been substantial research into activities during OSHC, and so the sector may have been fatigued from responding to surveys. In contrast, the enablers highlighted in this survey were consistent with the Delphi results, with staff training and educator understanding considered most important for guideline implementation. This formative work is important for to help ensure effective uptake in future trials [25].

Meeting the guideline criteria differed significantly by state and SES which has important implications for future implementation. State differences in practice may reflect state-level efforts that have been made in the OSHC sector. For example, Queensland and New South Wales have had state-level programs promoting PA in OSHC– PANOSH in Queensland [26], and “Eat Smart, Play Smart” in New South Wales[27]. Both these states were above the national average for meeting all guideline components, suggesting that these programs may be positively influencing practice. Further to this, Queensland experiences more average daily sunshine hours (range 7–11 h/day) than most other states and territories in Australia [28] – providing more opportunity for active outdoor play [29]. While the ACT and Tasmania also showed high rates of guideline compliance, only a small number of services from these jurisdictions participated in the study, reducing the confidence in the estimates. Unsurprisingly, there was a significant difference in the likelihood of high and low SES services meeting the guidelines, suggesting that interventions targeted in low SES services are warranted.

To our knowledge, this is the first national-level study of PA and ST practices in OSHC in any country. Importantly, it provided end-users the opportunity to comment on the new national OSHC-sector guidelines, which will help ensure feasibility and bolster the guidelines’ acceptance in the future. By examining how current practice meets new OSHC-sector guidelines, the study provides insight into the current evidence-practice gap. A further strength was representation from all states and territories.

Study limitations include the use of self-reported data, which is susceptible to social desirability bias [20]. In addition, the response rate was modest. We received anecdotal feedback that some OSHC directions did not receive the initial survey invitation (presumably due to spam filters). It is unclear how widespread this issue was since we cannot differentiate between non-respondents and those who didn’t receive the invitation. In addition, many OSHC services listed on the ACECQA database are run by large OSHC providers and shared a single centralised email address. It is unclear whether these email invitations ever reached the intended OSHC director recipients. Finally due to the survey length, other relevant topics, such as programming during vacation care, legislated workforce qualifications, costings, and venue requirements, could not be explored.

This study revealed there is a sizeable evidence-practice gap (i.e., that current practice does not meet evidence-based guidelines, particularly for ST in OSHC). The study also suggested that the new OSHC sector guidelines are well received by the sector, both in terms of the need and the specified durations of PA and ST recommended per care session. Concerted efforts will be needed to disseminate the guidelines to increase awareness of their existence, and familiarity with the content [30].

A range of strategies will be needed to achieve this. In particular, findings highlighted state level and SES difference, a need for OSHC sector staff training on how to use these guidelines, including children’s play behaviours, activities to encourage PA and reduce competing interests (i.e., avoiding offering screen activities concurrently with PA opportunities) [28, 29].

## Conclusion

In conclusion, this is the first national-level study examining daily scheduling for PA and ST in Australian OSHC. PA and ST guidelines were welcomed and considered feasible by the sector. More OSHC services are meeting the PA recommendations than ST recommendations, and only around 40% are meeting best-practice guidelines. Concerted efforts are now needed to work with the sector to implement the PA and ST guidelines into practice.

## Supplementary Information

**Additional file 1.**

**Additional file 2.**

**Additional file 3.**

**Additional file 4.**

**Additional file 5.**

## Data Availability

The datasets generated and/or analysed during the current study are not publicly due to the type of approval received from human research ethics committee at the University of South Australia; but they are available from the corresponding author on reasonable request.

## Abbreviations

ACECQA: Australian Children’s Education and Care Quality Authority
GRADE: Grading of Recommendations Assessment, Development and Evaluation
NOSHSA: National Outside School Hours Services Alliance
OSHC: Outside school hours care
PA: Physical activity
SEIFA: Socio-Economic Indexes for Areas
SES: Socioeconomic status
ST: Screen time
