# Supplementary material for: Physical activity and screen time in outside school hours care services across Australia: current versus best practice
Source: BMC Public Health. 2022 Apr 7;22:680. doi: 10.1186/s12889-022-13135-7 (PMC8991463; doi:10.1186/s12889-022-13135-7)
Supplement: Supplementary file 1 — Additional file 1. [file 12889_2022_13135_MOESM1_ESM.pdf]

Supplementary File 1: Australian physical activity and screen time guidelines for OSHC (16)

Session	Physical Activity	Screen Time*
**Before School Care**	Schedule **45 minutes** of time for children to engage in a variety of physical activities including energetic play. **More is better.**	Discourage children from engaging with screens, with total screen time **no more than 30 minutes.** **Less is better.**
**After School Care**	Schedule **90 minutes (1.5 hours)** for children to engage in a variety of physical activity, including energetic play . **More is better.**	Discourage children from engaging with screens, with total screen time **no more than 60 minutes.** **Less is better.**
**Vacation Care**	Throughout the day schedule at least **2-3 hours of time** for children to engage in a variety of physical activity, including energetic play . **More is better.**	Discourage children from engaging with screens. **No more than 2 hours** over the entire day. **Less is better.**

*does not include computer use for homework
