# Supplementary material for: Physical activity and screen time in outside school hours care services across Australia: current versus best practice
Source: BMC Public Health. 2022 Apr 7;22:680. doi: 10.1186/s12889-022-13135-7 (PMC8991463; doi:10.1186/s12889-022-13135-7)
Supplement: Supplementary file 2 — Additional file 2. [file 12889_2022_13135_MOESM2_ESM.pdf]

Supplementary File 2 – National Survey

Thank you for taking part in our survey *" Physical activity and Screen time practices in OSHC: Understanding Australian OSHC"*

We want to understand what types of activities and duration are offered in your OSHC service.

The following questions are asked across varying aspects of OSHC (before and after school care). If your service provides both, please complete both components, you will be prompted accordingly. The final section contains our draft Physical Activity and Screen Time Guidelines for OSHC for you to provide feedback and comment on. This survey should take approximately 15 minutes to complete.

1. Location:
   - ☐ Australian Capital Territory
   - ☐ New South Wales
   - ☐ Northern Territory
   - ☐ Queensland
   - ☐ South Australia
   - ☐ Tasmania
   - ☐ Victoria
   - ☐ Western Australia

2. What is your average daily attendance at OSHC?

3. Does your service provide Before School care? YES NO – if yes prompts to complete the components, if NO skips to AFTER SCHOOL CARE

4. What are your typical hours of operation?

Start Time:

End Time:

The following items will ask about general play, screen-based activities, and energetic play activities offered in your OSHC services during before school care.

These questions relate to your **TYPICAL** programming during before school care. Please think about what is offered on a typical day. If your schedule has changed due to COVID-19, please answer the questions based on what happened in your OSHC service **before COVID19**. (e.g. the first half of Term 1 2020).

For each activity, please indicate if it is available in 15 minute increments. Please only tick activities that are offered **every day or most** days.

For example, if a service offers energetic indoor play every day from 7:00 to 8:00, the form should be filled out like this:

Before School Care

* 7. Please select the times energetic play opportunities are available:

- [ ] 6:30 - 6:45am
- [ ] 6:45 - 7:00am
- [x] 7:00 - 7:15am
- [x] 7:15 - 7:30am
- [x] 7:30 - 7:45am
- [x] 7:45 - 8:00am
- [ ] 8:00 - 8:15am
- [ ] 8:15 - 8:30am

OK

If staff led active games are offered occasionally (e.g. once or twice a week), do not tick any boxes, as we are interested in what is happening **every day** or **most** days

5. Does your OSHC service provide opportunities for **Physical Activity** (e.g. sports equipment/playground play) during **Before School Care?**

If YES, prompts you to complete the questions, if NO skips to Recreational Screen Time

6. Do you provide energetic play opportunities indoors (e.g. indoor gym/hall/games)? If YES prompts to provide time, if NO skips to next question

7. Please select the times available for energetic play opportunities indoor:

**6:30 – 6.45am**	**6:45 – 7.00am**	**7:00 – 7:15am**	**7:15 – 7:30am**	**7:30 – 7:45am**	**7:45 – 8:00am**	**8:00 – 8:15am**	**8:00 – 8:15am**	**8:30 – 8:45am**	**8:45 – 9:00am**
○	○	○	○	○	○	○	○	○	○

8. Do you provide energetic play opportunities outdoors (e.g. oval/playground) ? If YES prompts to provide time, if NO skips to next question
9. Please select the time available:

**6:30 – 6.45am**	**6:45 – 7.00am**	**7:00 – 7:15am**	**7:15 – 7:30am**	**7:30 – 7:45am**	**7:45 – 8:00am**	**8:00 – 8:15am**	**8:00 – 8:15am**	**8:30 – 8:45am**	**8:45 – 9:00am**
○	○	○	○	○	○	○	○	○	○

10. Do you provide staff led active games/ activities? If YES prompts to provide time, if NO skips to next question

11. Please select the time available:

**6:30 – 6.45am**	**6:45 – 7.00am**	**7:00 – 7:15am**	**7:15 – 7:30am**	**7:30 – 7:45am**	**7:45 – 8:00am**	**8:00 – 8:15am**	**8:00 – 8:15am**	**8:30 – 8:45am**	**8:45 – 9:00am**
○	○	○	○	○	○	○	○	○	○

12. Can children access an indoor playground for active play? If YES prompts to provide time, if NO skips to next question

13. Please select the time available:

**6:30 – 6.45am**	**6:45 – 7.00am**	**7:00 – 7:15am**	**7:15 – 7:30am**	**7:30 – 7:45am**	**7:45 – 8:00am**	**8:00 – 8:15am**	**8:00 – 8:15am**	**8:30 – 8:45am**	**8:45 – 9:00am**
○	○	○	○	○	○	○	○	○	○

14. Can children access an outdoor playground for active play? If YES prompts to provide time, if NO skips to next question

15. Please select the time available:

**6:30 – 6.45am**	**6:45 – 7.00am**	**7:00 – 7:15am**	**7:15 – 7:30am**	**7:30 – 7:45am**	**7:45 – 8:00am**	**8:00 – 8:15am**	**8:00 – 8:15am**	**8:30 – 8:45am**	**8:45 – 9:00am**
○	○	○	○	○	○	○	○	○	○

16. Can children use sports equipment (e.g. balls/hoops) ? If YES prompts to provide time, if NO skips to next question
17. Please select the time available:

6:30 – 6.45am	6:45 – 7.00am	7:00 – 7:15am	7:15 – 7:30am	7:30 – 7:45am	7:45 – 8:00am	8:00 – 8:15am	8:00 – 8:15am	8:30 – 8:45am	8:45 – 9:00am
o	o	o	o	o	o	o	o	o	o

18. Does your service provide any other physically active activities not already listed during the before school care session? [List includes: energetic play indoors, energetic play outdoors, staff led games, playground inside, playground outside, sports equipment] If YES prompts to provide time and specify the activity, if NO skips to next question
19. Please select the time available and Specify the activity:

6:30 – 6.45am	6:45 – 7.00am	7:00 – 7:15am	7:15 – 7:30am	7:30 – 7:45am	7:45 – 8:00am	8:00 – 8:15am	8:00 – 8:15am	8:30 – 8:45am	8:45 – 9:00am
o	o	o	o	o	o	o	o	o	o

20. Does your service provide any other physically active activities not already listed during the before school care session? [List includes: energetic play indoors, energetic play outdoors, staff led games, playground inside, playground outside, sports equipment] If YES prompts to provide time and specify the activity, if NO skips to next question
21. Please select the time available and Specify the activity:

6:30 – 6.45am	6:45 – 7.00am	7:00 – 7:15am	7:15 – 7:30am	7:30 – 7:45am	7:45 – 8:00am	8:00 – 8:15am	8:00 – 8:15am	8:30 – 8:45am	8:45 – 9:00am
o	o	o	o	o	o	o	o	o	o

22. Does your service provide any other physically active activities not already listed during the before school care session? [List includes: energetic play indoors, energetic play outdoors, staff led games, playground inside, playground outside, sports equipment] If YES prompts to provide time and specify the activity, if NO skips to next question
23. Please select the time available and Specify the activity:

6:30 – 6.45am	6:45 – 7.00am	7:00 – 7:15am	7:15 – 7:30am	7:30 – 7:45am	7:45 – 8:00am	8:00 – 8:15am	8:00 – 8:15am	8:30 – 8:45am	8:45 – 9:00am
○	○	○	○	○	○	○	○	○	○

24. Does your OSHC service provide **Recreational Screen Time** (e.g. ipads/tv/computer) during **Before School Care?**

If YES, prompts you to complete the questions, if NO skips to Sedentary Activities

25. Do you allow TV/DVD viewing? If YES prompts to provide time, if NO skips to next question
26. Please select the time available:

6:30 – 6.45am	6:45 – 7.00am	7:00 – 7:15am	7:15 – 7:30am	7:30 – 7:45am	7:45 – 8:00am	8:00 – 8:15am	8:00 – 8:15am	8:30 – 8:45am	8:45 – 9:00am
○	○	○	○	○	○	○	○	○	○

27. Do you allow use of ipads/tablets? If YES prompts to provide time, if NO skips to next question
28. Please select the time available:

6:30 – 6.45am	6:45 – 7.00am	7:00 – 7:15am	7:15 – 7:30am	7:30 – 7:45am	7:45 – 8:00am	8:00 – 8:15am	8:00 – 8:15am	8:30 – 8:45am	8:45 – 9:00am
○	○	○	○	○	○	○	○	○	○

29. Do you allow use of computers (not for homework)? If YES prompts to provide time, if NO skips to next question
30. Please select the time available:

6:30 – 6.45am	6:45 – 7.00am	7:00 – 7:15am	7:15 – 7:30am	7:30 – 7:45am	7:45 – 8:00am	8:00 – 8:15am	8:00 – 8:15am	8:30 – 8:45am	8:45 – 9:00am
o	o	o	o	o	o	o	o	o	o

31. Do you allow access to seated video games? If YES prompts to provide time, if NO skips to next question
32. Please select the time available:

6:30 – 6.45am	6:45 – 7.00am	7:00 – 7:15am	7:15 – 7:30am	7:30 – 7:45am	7:45 – 8:00am	8:00 – 8:15am	8:00 – 8:15am	8:30 – 8:45am	8:45 – 9:00am
o	o	o	o	o	o	o	o	o	o

33. Do you allow access to standing/active video games? If YES prompts to provide time, if NO skips to next question
34. Please select the time available:

6:30 – 6.45am	6:45 – 7.00am	7:00 – 7:15am	7:15 – 7:30am	7:30 – 7:45am	7:45 – 8:00am	8:00 – 8:15am	8:00 – 8:15am	8:30 – 8:45am	8:45 – 9:00am
o	o	o	o	o	o	o	o	o	o

35. Does your service provide any other recreational screen activities not already listed ? [Listed: TV/DVD viewing, Computer Use, ipads/tablets, Video games seated, Video games standing] If YES prompts to provide time and specify activity, if NO skips to next question
36. Please select the time available:

6:30 – 6.45am	6:45 – 7.00am	7:00 – 7:15am	7:15 – 7:30am	7:30 – 7:45am	7:45 – 8:00am	8:00 – 8:15am	8:00 – 8:15am	8:30 – 8:45am	8:45 – 9:00am
o	o	o	o	o	o	o	o	o	o

37. Do you provide any other additional recreational screen activities? if YES prompts to provide time and specify activity, if NO skips to next question.
38. Please select the time available and SPECIFY the activity:

6:30 – 6.45am	6:45 – 7.00am	7:00 – 7:15am	7:15 – 7:30am	7:30 – 7:45am	7:45 – 8:00am	8:00 – 8:15am	8:00 – 8:15am	8:30 – 8:45am	8:45 – 9:00am
o	o	o	o	o	o	o	o	o	o

39. Do you provide any other additional recreational screen activities? if YES prompts to provide time and specify activity, if NO skips to next question.
40. Please select the time available and SPECIFY the activity:

6:30 – 6.45am	6:45 – 7.00am	7:00 – 7:15am	7:15 – 7:30am	7:30 – 7:45am	7:45 – 8:00am	8:00 – 8:15am	8:00 – 8:15am	8:30 – 8:45am	8:45 – 9:00am
o	o	o	o	o	o	o	o	o	o

41. Does your OSHC service provide **Sedentary Activities** (e.g. board games/lego/indoor toys) during **Before School Care?**

If YES, prompts you to complete the questions, if NO skips to After School Care
42. Does your service provide breakfast? If YES prompts to provide time, if NO skips to next question

43. Please select the time available:

6:30 – 6.45am	6:45 – 7.00am	7:00 – 7:15am	7:15 – 7:30am	7:30 – 7:45am	7:45 – 8:00am	8:00 – 8:15am	8:00 – 8:15am	8:30 – 8:45am	8:45 – 9:00am
○	○	○	○	○	○	○	○	○	○

44. Does your service provide arts and crafts? If YES prompts to provide time, if NO skips to next question

45. Please select the time available:

6:30 – 6.45am	6:45 – 7.00am	7:00 – 7:15am	7:15 – 7:30am	7:30 – 7:45am	7:45 – 8:00am	8:00 – 8:15am	8:00 – 8:15am	8:30 – 8:45am	8:45 – 9:00am
○	○	○	○	○	○	○	○	○	○

46. Does your service provide cooking/food preparation activities? If YES prompts to provide time, if NO skips to next question

47. Please select the time available:

6:30 – 6.45am	6:45 – 7.00am	7:00 – 7:15am	7:15 – 7:30am	7:30 – 7:45am	7:45 – 8:00am	8:00 – 8:15am	8:00 – 8:15am	8:30 – 8:45am	8:45 – 9:00am
○	○	○	○	○	○	○	○	○	○

48. Does your service provide board games / lego / construction activities? If YES prompts to provide time, if NO skips to next question

49. Please select the time available:

6:30 – 6.45am	6:45 – 7.00am	7:00 – 7:15am	7:15 – 7:30am	7:30 – 7:45am	7:45 – 8:00am	8:00 – 8:15am	8:00 – 8:15am	8:30 – 8:45am	8:45 – 9:00am
○	○	○	○	○	○	○	○	○	○

50. Does your service assist with homework/reading? If YES prompts to provide time, if NO skips to next question

51. Please select the time available:

6:30 – 6.45am	6:45 – 7.00am	7:00 – 7:15am	7:15 – 7:30am	7:30 – 7:45am	7:45 – 8:00am	8:00 – 8:15am	8:00 – 8:15am	8:30 – 8:45am	8:45 – 9:00am
○	○	○	○	○	○	○	○	○	○

52. Does your service provide time/space for children to do homework/reading **without** assistance? If YES prompts to provide time, if NO skips to next question
53. Please select the time available:

6:30 – 6.45am	6:45 – 7.00am	7:00 – 7:15am	7:15 – 7:30am	7:30 – 7:45am	7:45 – 8:00am	8:00 – 8:15am	8:00 – 8:15am	8:30 – 8:45am	8:45 – 9:00am
○	○	○	○	○	○	○	○	○	○

54. Does your service provide role play / dress up opportunities? If YES prompts to provide time, if NO skips to next question
55. Please select the time available:

6:30 – 6.45am	6:45 – 7.00am	7:00 – 7:15am	7:15 – 7:30am	7:30 – 7:45am	7:45 – 8:00am	8:00 – 8:15am	8:00 – 8:15am	8:30 – 8:45am	8:45 – 9:00am
○	○	○	○	○	○	○	○	○	○

56. Does your service provide opportunities to listen/play music? If YES prompts to provide time, if NO skips to next question
57. Please select the time available:

6:30 – 6.45am	6:45 – 7.00am	7:00 – 7:15am	7:15 – 7:30am	7:30 – 7:45am	7:45 – 8:00am	8:00 – 8:15am	8:00 – 8:15am	8:30 – 8:45am	8:45 – 9:00am
○	○	○	○	○	○	○	○	○	○

58. Does your service provide indoor freeplay? If YES prompts to provide time, if NO skips to next question

59. Please select the time available:

6:30 – 6.45am	6:45 – 7.00am	7:00 – 7:15am	7:15 – 7:30am	7:30 – 7:45am	7:45 – 8:00am	8:00 – 8:15am	8:00 – 8:15am	8:30 – 8:45am	8:45 – 9:00am
○	○	○	○	○	○	○	○	○	○

60. Does your service provide any other **sedentary activities** not already listed during the before school care session? [Listed: Breakfast, Arts/Crafts, Cooking/Food preparation, Boardgames/lego, Homework/reading with assistance, Homework/reading without assistance, Role play/dress ups, Listening to music/playing music, Indoor freeplay] If YES prompts to provide time and specify the activity, if NO skips to next question (AFTER SCHOOL CARE)
61. Please select the time available and specify the activity:

6:30 – 6.45am	6:45 – 7.00am	7:00 – 7:15am	7:15 – 7:30am	7:30 – 7:45am	7:45 – 8:00am	8:00 – 8:15am	8:00 – 8:15am	8:30 – 8:45am	8:45 – 9:00am
○	○	○	○	○	○	○	○	○	○

62. Does your service provide any other **sedentary activities** not already listed during the before school care session? [Listed: Breakfast, Arts/Crafts, Cooking/Food preparation, Boardgames/lego, Homework/reading with assistance, Homework/reading without assistance, Role play/dress ups, Listening to music/playing music, Indoor freeplay] If YES prompts to provide time and specify the activity, if NO skips to next question (AFTER SCHOOL CARE)
63. Please select the time available and specify the activity:

6:30 – 6.45am	6:45 – 7.00am	7:00 – 7:15am	7:15 – 7:30am	7:30 – 7:45am	7:45 – 8:00am	8:00 – 8:15am	8:00 – 8:15am	8:30 – 8:45am	8:45 – 9:00am
○	○	○	○	○	○	○	○	○	○

64. Does your service provide any other **sedentary activities** not already listed during the before school care session? [Listed: Breakfast, Arts/Crafts, Cooking/Food preparation, Boardgames/lego, Homework/reading with assistance, Homework/reading without

assistance, Role play/dress ups, Listening to music/playing music, Indoor freeplay] If YES prompts to provide time and specify the activity, if NO skips to next question (AFTER SCHOOL CARE)

65. Please select the time available and specify the activity:

**6:30 – 6.45am**	**6:45 – 7.00am**	**7:00 – 7:15am**	**7:15 – 7:30am**	**7:30 – 7:45am**	**7:45 – 8:00am**	**8:00 – 8:15am**	**8:00 – 8:15am**	**8:30 – 8:45am**	**8:45 – 9:00am**
○	○	○	○	○	○	○	○	○	○

66. Please provide any other comments about activity scheduling during Before School Care:

____

____

____

____

AFTER SCHOOL CARE

67. Does your OSHC service provide After School Care? If YES, prompts to next question, if NO Skips to Draft Guidelines.
68. What are your typical hours of operation?

The following questions relate to your **TYPICAL** programming during before school care. Please think about what is offered on a typical day. If you schedule has changed due to COVID-19, please answer the question based on what happened in your OSHC service **before COVID19**. (e.g. the first half of Term 1 2020).

For each activity, please indicate if it is available in 15 minute increments. Please only tick activities that are offered **every day or most** days.

For example, if a service offers energetic indoor play is offered every day from 3:305 to 5:30, the survey should be filled out like this:

* 9. Please select the times available for outdoor energetic play opportunities :

- [ ] 3:00 - 3:15pm
- [ ] 3:15 - 3:30pm
- [x] 3:30 - 3:45pm
- [x] 3:45 - 4:00pm
- [x] 4:00 - 4:15pm
- [x] 4:15 - 4:30pm
- [x] 4:30 - 4:45pm
- [x] 4:45 - 5:00pm
- [x] 5:00 - 5:15pm
- [x] 5:15 - 5:30pm
- [ ] 5:30 - 5:45pm
- [ ] 5:45 - 6:00pm
- [ ] 6:00 - 6:15pm
- [ ] 6:15 - 6:30pm

OK

If staff led active games are offered occasionally (e.g. once or twice a week), do not tick any boxes, as we are interested in what is happening **every day** or **most** days

69. Does your OSHC service provide **Physical Activity** (e.g. sports equipment, playground play) opportunities during **After School Care?** If YES prompts to next question, if NO prompts to **Recreational Screen Time.**

70. Do you provide energetic play opportunities indoor (e.g. indoor gym/hall/games)? If YES prompts to provide time, if NO skips to next question

71. Please select the time available:

3:00 – 3:15pm	3:15 – 3:30pm	3:30 – 3:45pm	3:45 – 4:00pm	4:00 – 4:15pm	4:15 – 4:30pm	4:30 – 4:45pm	4:45 – 5:00pm	5:00 – 5:15pm	5:15 – 5:30pm	5:30 – 5:45pm	5:45- 6:00pm	6:00 – 6:15pm	6:15 – 6:30pm
○	○	○	○	○	○	○	○	○	○	○	○	○	○

72. Do you provide energetic play opportunities outdoor (e.g. oval/playground)? If YES prompts to provide time, if NO skips to next question

73. Please select the time available:

3:00 – 3:15pm	3:15 – 3:30pm	3:30 – 3:45pm	3:45 – 4:00pm	4:00 – 4:15pm	4:15 – 4:30pm	4:30 – 4:45pm	4:45 – 5:00pm	5:00 – 5:15pm	5:15 – 5:30pm	5:30 – 5:45pm	5:45- 6:00pm	6:00 – 6:15pm	6:15 – 6:30pm
○	○	○	○	○	○	○	○	○	○	○	○	○	○

74. Do you provide staff led active games/active activities? If YES prompts to provide time, if NO skips to next question

75. Please select the time available:

3:00 – 3:15pm	3:15 – 3:30pm	3:30 – 3:45pm	3:45 – 4:00pm	4:00 – 4:15pm	4:15 – 4:30pm	4:30 – 4:45pm	4:45 – 5:00pm	5:00 – 5:15pm	5:15 – 5:30pm	5:30 – 5:45pm	5:45- 6:00pm	6:00 – 6:15pm	6:15 – 6:30pm
○	○	○	○	○	○	○	○	○	○	○	○	○	○

76. Can children access an indoor playground for active play? If YES prompts to provide time, if NO skips to next question
77. Please select the time available:

**3:00 – 3:15pm**	**3:15 – 3:30pm**	**3:30 – 3:45pm**	**3:45 – 4:00pm**	**4:00 – 4:15pm**	**4:15 – 4:30pm**	**4:30 – 4:45pm**	**4:45 – 5:00pm**	**5:00 – 5:15pm**	**5:15 – 5:30pm**	**5:30 – 5:45pm**	**5:45- 6:00pm**	**6:00 – 6:15pm**	**6:15 – 6:30pm**
○	○	○	○	○	○	○	○	○	○	○	○	○	○

78. Can children access an outdoor playground for active play? If YES prompts to provide time, if NO skips to next question
79. Please select the time available:

**3:00 – 3:15pm**	**3:15 – 3:30pm**	**3:30 – 3:45pm**	**3:45 – 4:00pm**	**4:00 – 4:15pm**	**4:15 – 4:30pm**	**4:30 – 4:45pm**	**4:45 – 5:00pm**	**5:00 – 5:15pm**	**5:15 – 5:30pm**	**5:30 – 5:45pm**	**5:45- 6:00pm**	**6:00 – 6:15pm**	**6:15 – 6:30pm**
○	○	○	○	○	○	○	○	○	○	○	○	○	○

80. Can children use sports equipment (e.g. balls/hoops)? If YES prompts to provide time, if NO skips to next question
81. Please select the time available:

**3:00 – 3:15pm**	**3:15 – 3:30pm**	**3:30 – 3:45pm**	**3:45 – 4:00pm**	**4:00 – 4:15pm**	**4:15 – 4:30pm**	**4:30 – 4:45pm**	**4:45 – 5:00pm**	**5:00 – 5:15pm**	**5:15 – 5:30pm**	**5:30 – 5:45pm**	**5:45- 6:00pm**	**6:00 – 6:15pm**	**6:15 – 6:30pm**
○	○	○	○	○	○	○	○	○	○	○	○	○	○

82. Does your service provide any other physical activities not already listed during the afterschool care session? [List includes: energetic play indoors, energetic play outdoors, staff led games, playground inside, playground outside, sports equipment] If YES prompts to provide time and specify the activity, if NO skips to next question
83. Please select the time available and Specify the activity:

3:00 – 3:15pm	3:15 – 3:30pm	3:30 – 3:45pm	3:45 – 4:00pm	4:00 – 4:15pm	4:15 – 4:30pm	4:30 – 4:45pm	4:45 – 5:00pm	5:00 – 5:15pm	5:15 – 5:30pm	5:30 – 5:45pm	5:45- 6:00pm	6:00 – 6:15pm	6:15 – 6:30pm
○	○	○	○	○	○	○	○	○	○	○	○	○	○

84. Does your service provide any other physical activities not already listed during the afterschool care session? [List includes: energetic play indoors, energetic play outdoors, staff led games, playground inside, playground outside, sports equipment] If YES prompts to provide time and specify the activity, if NO skips to next question
85. Please select the time available and Specify the activity:

3:00 – 3:15pm	3:15 – 3:30pm	3:30 – 3:45pm	3:45 – 4:00pm	4:00 – 4:15pm	4:15 – 4:30pm	4:30 – 4:45pm	4:45 – 5:00pm	5:00 – 5:15pm	5:15 – 5:30pm	5:30 – 5:45pm	5:45- 6:00pm	6:00 – 6:15pm	6:15 – 6:30pm
○	○	○	○	○	○	○	○	○	○	○	○	○	○

86. Does your service provide any other physical activities not already listed during the afterschool care session? [List includes: energetic play indoors, energetic play outdoors, staff led games, playground inside, playground outside, sports equipment] If YES prompts to provide time and specify the activity, if NO skips to next question
87. Please select the time available and Specify the activity:

3:00 – 3:15pm	3:15 – 3:30pm	3:30 – 3:45pm	3:45 – 4:00pm	4:00 – 4:15pm	4:15 – 4:30pm	4:30 – 4:45pm	4:45 – 5:00pm	5:00 – 5:15pm	5:15 – 5:30pm	5:30 – 5:45pm	5:45- 6:00pm	6:00 – 6:15pm	6:15 – 6:30pm
○	○	○	○	○	○	○	○	○	○	○	○	○	○

88. Does your OSHC service provide **Recreational Screen Time** (e.g. TV/DVD, computer use) opportunities during **After School Care?**

If YES, prompts you to complete the questions, if NO skips to Sedentary Activities

89. Do you allow TV/DVD viewing? If YES prompts to provide time, if NO skips to next question
90. Please select the time available:

3:00 – 3:15pm	3:15 – 3:30pm	3:30 – 3:45pm	3:45 – 4:00pm	4:00 – 4:15pm	4:15 – 4:30pm	4:30 – 4:45pm	4:45 – 5:00pm	5:00 – 5:15pm	5:15 – 5:30pm	5:30 – 5:45pm	5:45- 6:00pm	6:00 – 6:15pm	6:15 – 6:30pm
○	○	○	○	○	○	○	○	○	○	○	○	○	○

91. Do you allow use of ipads/tablets? If YES prompts to provide time, if NO skips to next question
92. Please select the time available:

3:00 – 3:15pm	3:15 – 3:30pm	3:30 – 3:45pm	3:45 – 4:00pm	4:00 – 4:15pm	4:15 – 4:30pm	4:30 – 4:45pm	4:45 – 5:00pm	5:00 – 5:15pm	5:15 – 5:30pm	5:30 – 5:45pm	5:45- 6:00pm	6:00 – 6:15pm	6:15 – 6:30pm
○	○	○	○	○	○	○	○	○	○	○	○	○	○

93. Do you allow use of computers (not for homework)? If YES prompts to provide time, if NO skips to next question
94. Please select the time available:

3:00 – 3:15pm	3:15 – 3:30pm	3:30 – 3:45pm	3:45 – 4:00pm	4:00 – 4:15pm	4:15 – 4:30pm	4:30 – 4:45pm	4:45 – 5:00pm	5:00 – 5:15pm	5:15 – 5:30pm	5:30 – 5:45pm	5:45- 6:00pm	6:00 – 6:15pm	6:15 – 6:30pm
○	○	○	○	○	○	○	○	○	○	○	○	○	○

95. Do you allow access to seated video games? If YES prompts to provide time, if NO skips to next question
96. Please select the time available:

3:00 – 3:15pm	3:15 – 3:30pm	3:30 – 3:45pm	3:45 – 4:00pm	4:00 – 4:15pm	4:15 – 4:30pm	4:30 – 4:45pm	4:45 – 5:00pm	5:00 – 5:15pm	5:15 – 5:30pm	5:30 – 5:45pm	5:45- 6:00pm	6:00 – 6:15pm	6:15 – 6:30pm
○	○	○	○	○	○	○	○	○	○	○	○	○	○

97. Do you allow access to standing/active video games? If YES prompts to provide time, if NO skips to next question
98. Please select the time available:

3:00 – 3:15pm	3:15 – 3:30pm	3:30 – 3:45pm	3:45 – 4:00pm	4:00 – 4:15pm	4:15 – 4:30pm	4:30 – 4:45pm	4:45 – 5:00pm	5:00 – 5:15pm	5:15 – 5:30pm	5:30 – 5:45pm	5:45- 6:00pm	6:00 – 6:15pm	6:15 – 6:30pm
○	○	○	○	○	○	○	○	○	○	○	○	○	○

99. Does your service provide any other recreational screen activities not already listed ? [Listed: TV/DVD viewing, Computer Use, ipads/tablets, Video games seated, Video games standing] If YES prompts to provide time, if NO skips to next question
100. Please select the time available:

3:00 – 3:15pm	3:15 – 3:30pm	3:30 – 3:45pm	3:45 – 4:00pm	4:00 – 4:15pm	4:15 – 4:30pm	4:30 – 4:45pm	4:45 – 5:00pm	5:00 – 5:15pm	5:15 – 5:30pm	5:30 – 5:45pm	5:45- 6:00pm	6:00 – 6:15pm	6:15 – 6:30pm
○	○	○	○	○	○	○	○	○	○	○	○	○	○

101. Does your service provide any other recreational screen activities not already listed ? [Listed: TV/DVD viewing, Computer Use, ipads/tablets, Video games seated, Video games standing] If YES prompts to provide time and specify the activity, if NO skips to next question
102. Please select the time available and specify the activity:

3:00 – 3:15pm	3:15 – 3:30pm	3:30 – 3:45pm	3:45 – 4:00pm	4:00 – 4:15pm	4:15 – 4:30pm	4:30 – 4:45pm	4:45 – 5:00pm	5:00 – 5:15pm	5:15 – 5:30pm	5:30 – 5:45pm	5:45-6:00pm	6:00 – 6:15pm	6:15 – 6:30pm
○	○	○	○	○	○	○	○	○	○	○	○	○	○

103. Does your service provide any other recreational screen activities not already listed ? [Listed: TV/DVD viewing, Computer Use, ipads/tablets, Video games seated, Video games standing] If YES prompts to provide time and specify the activity, if NO skips to next question

104. Please select the time available and specify the activity:

3:00 – 3:15pm	3:15 – 3:30pm	3:30 – 3:45pm	3:45 – 4:00pm	4:00 – 4:15pm	4:15 – 4:30pm	4:30 – 4:45pm	4:45 – 5:00pm	5:00 – 5:15pm	5:15 – 5:30pm	5:30 – 5:45pm	5:45-6:00pm	6:00 – 6:15pm	6:15 – 6:30pm
○	○	○	○	○	○	○	○	○	○	○	○	○	○

105. Does your OSHC service provide **Sedentary Activities** (e.g. board games/lego/indoor toys) during **After School Care?** If YES, prompts you to complete the questions, if NO skips to After School Care

106. Does your service provide arts and crafts? If YES prompts to provide time, if NO skips to next question

107. Please select the time available:

3:00 – 3:15pm	3:15 – 3:30pm	3:30 – 3:45pm	3:45 – 4:00pm	4:00 – 4:15pm	4:15 – 4:30pm	4:30 – 4:45pm	4:45 – 5:00pm	5:00 – 5:15pm	5:15 – 5:30pm	5:30 – 5:45pm	5:45-6:00pm	6:00 – 6:15pm	6:15 – 6:30pm
○	○	○	○	○	○	○	○	○	○	○	○	○	○

108. Does your service provide cooking/food preparation activities? If YES prompts to provide time, if NO skips to next question

109. Please select the time available:

3:00 – 3:15pm	3:15 – 3:30pm	3:30 – 3:45pm	3:45 – 4:00pm	4:00 – 4:15pm	4:15 – 4:30pm	4:30 – 4:45pm	4:45 – 5:00pm	5:00 – 5:15pm	5:15 – 5:30pm	5:30 – 5:45pm	5:45-6:00pm	6:00 – 6:15pm	6:15 – 6:30pm

○	○	○	○	○	○	○	○	○	○	○	○	○	○

110. Does your service provide board games / lego / construction activities? If YES prompts to provide time, if NO skips to next question
111. Please select the time available:

**3:00 – 3:15pm**	**3:15 – 3:30pm**	**3:30 – 3:45pm**	**3:45 – 4:00pm**	**4:00 – 4:15pm**	**4:15 – 4:30pm**	**4:30 – 4:45pm**	**4:45 – 5:00pm**	**5:00 – 5:15pm**	**5:15 – 5:30pm**	**5:30 – 5:45pm**	**5:45- 6:00pm**	**6:00 – 6:15pm**	**6:15 – 6:30pm**
○	○	○	○	○	○	○	○	○	○	○	○	○	○

112. Does your service assist with homework/reading? If YES prompts to provide time, if NO skips to next question
113. Please select the time available:

**3:00 – 3:15pm**	**3:15 – 3:30pm**	**3:30 – 3:45pm**	**3:45 – 4:00pm**	**4:00 – 4:15pm**	**4:15 – 4:30pm**	**4:30 – 4:45pm**	**4:45 – 5:00pm**	**5:00 – 5:15pm**	**5:15 – 5:30pm**	**5:30 – 5:45pm**	**5:45- 6:00pm**	**6:00 – 6:15pm**	**6:15 – 6:30pm**
○	○	○	○	○	○	○	○	○	○	○	○	○	○

114. Does your service provide time/space for children to do homework/reading **without** assistance? If YES prompts to provide time, if NO skips to next question
115. Please select the time available:

**3:00 – 3:15pm**	**3:15 – 3:30pm**	**3:30 – 3:45pm**	**3:45 – 4:00pm**	**4:00 – 4:15pm**	**4:15 – 4:30pm**	**4:30 – 4:45pm**	**4:45 – 5:00pm**	**5:00 – 5:15pm**	**5:15 – 5:30pm**	**5:30 – 5:45pm**	**5:45- 6:00pm**	**6:00 – 6:15pm**	**6:15 – 6:30pm**
○	○	○	○	○	○	○	○	○	○	○	○	○	○

116. Does your service provide role play / dress up opportunities? If YES prompts to provide time, if NO skips to next question
117. Please select the time available:

3:00 – 3:15pm	3:15 – 3:30pm	3:30 – 3:45pm	3:45 – 4:00pm	4:00 – 4:15pm	4:15 – 4:30pm	4:30 – 4:45pm	4:45 – 5:00pm	5:00 – 5:15pm	5:15 – 5:30pm	5:30 – 5:45pm	5:45- 6:00pm	6:00 – 6:15pm	6:15 – 6:30pm
○	○	○	○	○	○	○	○	○	○	○	○	○	○

118. Does your service provide opportunities to listen/play music? If YES prompts to provide time, if NO skips to next question
119. Please select the time available:

3:00 – 3:15pm	3:15 – 3:30pm	3:30 – 3:45pm	3:45 – 4:00pm	4:00 – 4:15pm	4:15 – 4:30pm	4:30 – 4:45pm	4:45 – 5:00pm	5:00 – 5:15pm	5:15 – 5:30pm	5:30 – 5:45pm	5:45- 6:00pm	6:00 – 6:15pm	6:15 – 6:30pm
○	○	○	○	○	○	○	○	○	○	○	○	○	○

120. Does your service provide indoor freeplay? If YES prompts to provide time, if NO skips to next question
121. Please select the time available:

3:00 – 3:15pm	3:15 – 3:30pm	3:30 – 3:45pm	3:45 – 4:00pm	4:00 – 4:15pm	4:15 – 4:30pm	4:30 – 4:45pm	4:45 – 5:00pm	5:00 – 5:15pm	5:15 – 5:30pm	5:30 – 5:45pm	5:45- 6:00pm	6:00 – 6:15pm	6:15 – 6:30pm
○	○	○	○	○	○	○	○	○	○	○	○	○	○

122. Does your service provide any other **sedentary activities** not already listed during the after school care session? [Listed: Breakfast, Arts/Crafts, Cooking/Food preparation, Boardgames/lego, Homework/reading with assistance, Homework/reading without assistance, Role play/dress ups, Listening to music/playing music, Indoor freeplay] If YES prompts to provide time, if NO skips to next question (Draft Guidelines)
123. Please select the time available and specify the activity:

3:00 – 3:15pm	3:15 – 3:30pm	3:30 – 3:45pm	3:45 – 4:00pm	4:00 – 4:15pm	4:15 – 4:30pm	4:30 – 4:45pm	4:45 – 5:00pm	5:00 – 5:15pm	5:15 – 5:30pm	5:30 – 5:45pm	5:45- 6:00pm	6:00 – 6:15pm	6:15 – 6:30pm
○	○	○	○	○	○	○	○	○	○	○	○	○	○

124. Does your service provide any other **sedentary activities** not already listed during the after school care session? [Listed: Breakfast, Arts/Crafts, Cooking/Food preparation, Boardgames/lego, Homework/reading with assistance, Homework/reading without assistance, Role play/dress ups, Listening to music/playing music, Indoor freeplay] If YES prompts to provide time, if NO skips to next question (Draft Guidelines)

125. please select the time available and specify the activity:

3:00 – 3:15pm	3:15 – 3:30pm	3:30 – 3:45pm	3:45 – 4:00pm	4:00 – 4:15pm	4:15 – 4:30pm	4:30 – 4:45pm	4:45 – 5:00pm	5:00 – 5:15pm	5:15 – 5:30pm	5:30 – 5:45pm	5:45- 6:00pm	6:00 – 6:15pm	6:15 – 6:30pm
○	○	○	○	○	○	○	○	○	○	○	○	○	○

126. Does your service provide any other **sedentary activities** not already listed during the after school care session? [Listed: Breakfast, Arts/Crafts, Cooking/Food preparation, Boardgames/lego, Homework/reading with assistance, Homework/reading without assistance, Role play/dress ups, Listening to music/playing music, Indoor freeplay] If YES prompts to provide time, if NO skips to next question (Draft Guidelines)

127. please select the time available and specify the activity:

3:00 – 3:15pm	3:15 – 3:30pm	3:30 – 3:45pm	3:45 – 4:00pm	4:00 – 4:15pm	4:15 – 4:30pm	4:30 – 4:45pm	4:45 – 5:00pm	5:00 – 5:15pm	5:15 – 5:30pm	5:30 – 5:45pm	5:45- 6:00pm	6:00 – 6:15pm	6:15 – 6:30pm
○	○	○	○	○	○	○	○	○	○	○	○	○	○

128. Please provide any other comments about activity scheduling during After School Care:

____
____
____
____

DRAFT GUIDELINES

The following are guidelines developed by our team through a stakeholder consultation process. In this process we surveyed several OSHC educators, parents, researchers, health professional and academics and combined this with the best available evidence to provide these guidelines as a suggestion for how to improve physical activity and screen time practices in OSHC

**Session**	**Physical Activity**	**Recreational Screen Time***
**Before School Care**	Schedule **45 minutes** of time for children to engage in a variety of physical activities including energetic play. **More is better.**	Discourage children from engaging with screens, with total screen time no more than **30 minutes. Less is better.**
**After School Care**	Schedule **90 minutes** of time for children to engage in a variety of physical activities, including energetic play. **More is better.**	Discourage children from engaging with screens, with total screen time no more than **60 minutes. Less is better.**
**Vacation Care**	Throughout the day schedule **at least 3 hours** of time for children to engage in a variety of physical activities, including energetic play. **More is better.**	Discourage children from engaging with screens. No more than **2 hours** over the entire day. **Less is better.**

After having read these guidelines, what are your overall thoughts/opinions/comments?

Please select the comment you agree with most:

129. For Before School Care, the physical activity recommendation is:
   a. too high
   b. just right
   c. too low

Please explain your answer:

____

____

130. For After School care, the physical activity recommendation is:
   a) too high
   b) just right
   c) too low

Please explain your answer:

____

____

131. For Vacation Care, the physical activity recommendation is:

a) too high
b) just right
c) too low

Please explain your answer:

____

____

132. Do you have any additional comments with regard to the physical activity time recommendations in any aspect of OSHC?

____

____

Please select the comment you agree with most:

133. For Before School Care, the screen time recommendation is:

a. too high
b. just right
c. too low

Please explain your answer:

____

____

134. For After School care, the screen time recommendation is:
   d) too high
   e) just right
   f) too low

Please explain your answer:

____

____

135. For Vacation Care, the screen time recommendation is:
   d) too high
   e) just right
   f) too low

Please explain your answer:

____

____

136. Do you have any additional comments with regard to the screen time recommendation in any aspect of OSHC?:

____

____

137. Please provide feedback on the **wording** of the physical activity recommendations:

____

____

138. Please provide feedback on the **wording** of the screen time recommendations:

____

____

139. How confident are you that you could adjust your programming to suit these physical activity recommendations? (1 not at all, 5 very confident)

Not at all	Not confident	Neutral	Confident	Very confident
1	2	3	4	5

Please justify your answer (long text):

____

____

____

140. How confident are you that you could adjust your programming to suit these recreational screen time recommendations? (1 not at all, 5 very confident)

Not at all	Not confident	Neutral	Confident	Very confident
1	2	3	4	5

Please justify your answer(long text):

____

____

____

141. What would be the most appropriate way to communicate the guidelines and recommendations to staff? (select all that apply)

- ○ Face to face workshop
- ○ Online workshop
- ○ Video Tutorial
- ○ From a supervisor at work
- ○ Written material to read in own time
- ○ Combination of all the above

140. Do you have any additional comments/suggestions?

____
____
____

141. What duration of session length is appropriate for this staff training?

- o 30 mins
- o 60 mins
- o 2 hours
- o 3 hours

142. What types of educational/training materials would be appropriate for staff and the OSHC centre to help implement the guidelines? (select all that apply)

- o Online training
- o Electronic resources
- o Printed resources for staff (posters, cards, folders)
- o Printed resources for children (posters, cards, folders)
- o Other please specify:

143. Please comment on what is important to consider as part of staff training (open, long text):

____

____

____

144. Please rank what you consider to be the top 3 barriers to implementing these guidelines:

- ○ Indoor play space
- ○ Behaviour management
- ○ Family attitudes
- ○ Children’s attitudes
- ○ Staffing beliefs
- ○ Workplace culture
- ○ Other

145. Please rank what you consider to be the top 3 enablers to implementing these guidelines:

- ○ Adequate and appropriate training for who?
- ○ Staff understanding of the guidelines
- ○ OSHC families education and understanding of the guidelines
- ○ Workplace policy
- ○ Other
