# Supplementary material for: Physical activity and screen time in outside school hours care services across Australia: current versus best practice
Source: BMC Public Health. 2022 Apr 7;22:680. doi: 10.1186/s12889-022-13135-7 (PMC8991463; doi:10.1186/s12889-022-13135-7)
Supplement: Supplementary file 3 — Additional file 3. [file 12889_2022_13135_MOESM3_ESM.pdf]

Supplementary File 3: Post Hoc Testing for Physical Activity and Screen Time Scheduling

ONE WAY ANOVA ASSESSMENT OF PA TIME SCHEDULED BY SES TERTILE

ANOVA

		Sum of Squares	df	Mean Square	F	Sig.
BSC_totalPA	Between Groups	3980.841	2	1990.420	1.581	.207
	Within Groups	463326.572	368	1259.040		
	Total	467307.412	370			
ASC_totalPA	Between Groups	5539.342	2	2769.671	1.908	.150
	Within Groups	528442.675	364	1451.766		
	Total	533982.016	366			

Multiple Comparisons

Tukey HSD

Dependent Variable	(I) SES_Index	(J) SES_Index	Mean Difference (I-J)	Std. Error	Sig.	95% Confidence Interval Lower Bound	Upper Bound
BSC_totalPA	1.00	2.00	-7.76996	4.86309	.248	-19.2140	3.6740
		3.00	-6.41786	4.32904	.300	-16.6051	3.7694
	2.00	1.00	7.76996	4.86309	.248	-3.6740	19.2140
		3.00	1.35210	4.58809	.953	-9.4448	12.1490
	3.00	1.00	6.41786	4.32904	.300	-3.7694	16.6051
		2.00	-1.35210	4.58809	.953	-12.1490	9.4448
ASC_totalPA	1.00	2.00	-7.49823	5.10381	.307	-19.5092	4.5128
		3.00	-8.46834	4.63967	.163	-19.3871	2.4504
	2.00	1.00	7.49823	5.10381	.307	-4.5128	19.5092
		3.00	-.97011	5.04093	.980	-12.8331	10.8929
	3.00	1.00	8.46834	4.63967	.163	-2.4504	19.3871
		2.00	.97011	5.04093	.980	-10.8929	12.8331

ONE WAY ANOVA ASSESSMENT OF PA TIME SCHEDULED BY STATE

ANOVA

		Sum of Squares	df	Mean Square	F	Sig.
BSC_totalPA	Between Groups	34641.673	7	4948.810	3.903	.000
	Within Groups	530023.820	418	1268.000		
	Total	564665.493	425			
ASC_totalPA	Between Groups	33527.056	7	4789.579	3.311	.002
	Within Groups	601719.583	416	1446.441		
	Total	635246.639	423			

Multiple Comparisons

Tukey HSD

Dependent Variable	(I) state of Australia	(J) state of Australia	Mean Difference (I-J)	Std. Error	Sig.	95% Confidence Interval Lower Bound	95% Confidence Interval Upper Bound
BSC_totalPA	Australian Capital Territory	New South Wales	-8.58000	14.88211	.999	-53.9161	36.7561
		Northern Territory	16.50000	21.56231	.995	-49.1864	82.1864
		Queensland	-28.14706	15.04164	.572	-73.9692	17.6751
		South Australia	-5.66038	14.94308	1.000	-51.1822	39.8615
		Tasmania	-11.25000	19.23105	.999	-69.8345	47.3345
		Victoria	-9.46721	15.23549	.999	-55.8799	36.9454
		Western Australia	-4.50000	15.92482	1.000	-53.0126	44.0126
	New South Wales	Australian Capital Territory	8.58000	14.88211	.999	-36.7561	53.9161
		Northern Territory	25.08000	16.24019	.783	-24.3933	74.5533
		Queensland	-19.56706*	5.00616	.003	-34.8176	-4.3165
		South Australia	2.91962	4.70173	.999	-11.4035	17.2427
		Tasmania	-2.67000	12.98630	1.000	-42.2308	36.8908
		Victoria	-.88721	5.56155	1.000	-17.8297	16.0552
		Western Australia	4.08000	7.23952	.999	-17.9741	26.1341
	Northern Territory	Australian Capital Territory	-16.50000	21.56231	.995	-82.1864	49.1864
		New South Wales	-25.08000	16.24019	.783	-74.5533	24.3933
		Queensland	-44.64706	16.38651	.118	-94.5661	5.2720
		South Australia	-22.16038	16.29608	.875	-71.8040	27.4832
		Tasmania	-27.75000	20.30024	.872	-89.5917	34.0917

		Victoria	-25.96721	16.56462	.769	-76.4289	24.4945
		Western Australia	-21.00000	17.20077	.925	-73.3996	31.3996
	Queensland	Australian Capital Territory	28.14706	15.04164	.572	-17.6751	73.9692
		New South Wales	19.56706*	5.00616	.003	4.3165	34.8176
		Northern Territory	44.64706	16.38651	.118	-5.2720	94.5661
		South Australia	22.48668*	5.18458	.000	6.6926	38.2807
		Tasmania	16.89706	13.16881	.905	-23.2198	57.0139
		Victoria	18.67985*	5.97533	.040	.4769	36.8828
		Western Australia	23.64706*	7.56203	.039	.6105	46.6837
	South Australia	Australian Capital Territory	5.66038	14.94308	1.000	-39.8615	51.1822
		New South Wales	-2.91962	4.70173	.999	-17.2427	11.4035
		Northern Territory	22.16038	16.29608	.875	-27.4832	71.8040
		Queensland	-22.48668*	5.18458	.000	-38.2807	-6.6926
		Tasmania	-5.58962	13.05612	1.000	-45.3631	34.1839
		Victoria	-3.80684	5.72269	.998	-21.2402	13.6265
		Western Australia	1.16038	7.36403	1.000	-21.2730	23.5938
	Tasmania	Australian Capital Territory	11.25000	19.23105	.999	-47.3345	69.8345
		New South Wales	2.67000	12.98630	1.000	-36.8908	42.2308
		Northern Territory	27.75000	20.30024	.872	-34.0917	89.5917
		Queensland	-16.89706	13.16881	.905	-57.0139	23.2198
		South Australia	5.58962	13.05612	1.000	-34.1839	45.3631
		Victoria	1.78279	13.38980	1.000	-39.0073	42.5728
		Western Australia	6.75000	14.16921	1.000	-36.4144	49.9144

	Victoria	Australian Capital Territory	9.46721	15.23549	.999	-36.9454	55.8799
		New South Wales	.88721	5.56155	1.000	-16.0552	17.8297
		Northern Territory	25.96721	16.56462	.769	-24.4945	76.4289
		Queensland	-18.67985*	5.97533	.040	-36.8828	-.4769
		South Australia	3.80684	5.72269	.998	-13.6265	21.2402
		Tasmania	-1.78279	13.38980	1.000	-42.5728	39.0073
		Western Australia	4.96721	7.94063	.999	-19.2227	29.1572
	Western Australia	Australian Capital Territory	4.50000	15.92482	1.000	-44.0126	53.0126
		New South Wales	-4.08000	7.23952	.999	-26.1341	17.9741
		Northern Territory	21.00000	17.20077	.925	-31.3996	73.3996
		Queensland	-23.64706*	7.56203	.039	-46.6837	-.6105
		South Australia	-1.16038	7.36403	1.000	-23.5938	21.2730
		Tasmania	-6.75000	14.16921	1.000	-49.9144	36.4144
		Victoria	-4.96721	7.94063	.999	-29.1572	19.2227
ASC_totalPA	Australian Capital Territory	New South Wales	23.34677	13.87336	.698	-18.9174	65.6109
		Northern Territory	31.60714	19.68349	.747	-28.3571	91.5714
		Queensland	26.43293	14.08704	.568	-16.4822	69.3481
		South Australia	31.52228	13.96876	.320	-11.0325	74.0771
		Tasmania	-23.75000	20.53969	.944	-86.3226	38.8226
		Victoria	37.10821	14.22652	.156	-6.2318	80.4483
		Western Australia	19.78448	15.18823	.898	-26.4854	66.0543
	New South Wales	Australian Capital Territory	-23.34677	13.87336	.698	-65.6109	18.9174
		Northern Territory	8.26037	14.77496	.999	-36.7504	53.2712

		Queensland	3.08615	5.41335	.999	-13.4052	19.5775
		South Australia	8.17550	5.09765	.748	-7.3541	23.7051
		Tasmania	-47.09677	15.89775	.063	-95.5281	1.3345
		Victoria	13.76143	5.76659	.251	-3.8060	31.3289
		Western Australia	-3.56229	7.84488	1.000	-27.4611	20.3366
	Northern Territory	Australian Capital Territory	-31.60714	19.68349	.747	-91.5714	28.3571
		New South Wales	-8.26037	14.77496	.999	-53.2712	36.7504
		Queensland	-5.17422	14.97578	1.000	-50.7968	40.4484
		South Australia	-.08487	14.86458	1.000	-45.3687	45.1990
		Tasmania	-55.35714	21.15911	.153	-119.8168	9.1025
		Victoria	5.50107	15.10706	1.000	-40.5215	51.5236
		Western Australia	-11.82266	16.01598	.996	-60.6142	36.9689
	Queensland	Australian Capital Territory	-26.43293	14.08704	.568	-69.3481	16.4822
		New South Wales	-3.08615	5.41335	.999	-19.5775	13.4052
		Northern Territory	5.17422	14.97578	1.000	-40.4484	50.7968
		South Australia	5.08935	5.65338	.986	-12.1333	22.3120
		Tasmania	-50.18293*	16.08456	.040	-99.1834	-1.1825
		Victoria	10.67528	6.26324	.684	-8.4052	29.7558
		Western Australia	-6.64844	8.21686	.993	-31.6805	18.3836
	South Australia	Australian Capital Territory	-31.52228	13.96876	.320	-74.0771	11.0325
		New South Wales	-8.17550	5.09765	.748	-23.7051	7.3541
		Northern Territory	.08487	14.86458	1.000	-45.1990	45.3687
		Queensland	-5.08935	5.65338	.986	-22.3120	12.1333

		Tasmania	-55.27228*	15.98107	.014	-103.9574	-6.5871
		Victoria	5.58593	5.99249	.983	-12.6697	23.8416
		Western Australia	-11.73779	8.01240	.826	-36.1470	12.6714
	Tasmania	Australian Capital Territory	23.75000	20.53969	.944	-38.8226	86.3226
		New South Wales	47.09677	15.89775	.063	-1.3345	95.5281
		Northern Territory	55.35714	21.15911	.153	-9.1025	119.8168
		Queensland	50.18293*	16.08456	.040	1.1825	99.1834
		South Australia	55.27228*	15.98107	.014	6.5871	103.9574
		Victoria	60.85821*	16.20686	.005	11.4852	110.2312
		Western Australia	43.53448	17.05728	.177	-8.4293	95.4982
	Victoria	Australian Capital Territory	-37.10821	14.22652	.156	-80.4483	6.2318
		New South Wales	-13.76143	5.76659	.251	-31.3289	3.8060
		Northern Territory	-5.50107	15.10706	1.000	-51.5236	40.5215
		Queensland	-10.67528	6.26324	.684	-29.7558	8.4052
		South Australia	-5.58593	5.99249	.983	-23.8416	12.6697
		Tasmania	-60.85821*	16.20686	.005	-110.2312	-11.4852
		Western Australia	-17.32373	8.45375	.450	-43.0775	8.4300
	Western Australia	Australian Capital Territory	-19.78448	15.18823	.898	-66.0543	26.4854
		New South Wales	3.56229	7.84488	1.000	-20.3366	27.4611
		Northern Territory	11.82266	16.01598	.996	-36.9689	60.6142
		Queensland	6.64844	8.21686	.993	-18.3836	31.6805
		South Australia	11.73779	8.01240	.826	-12.6714	36.1470
		Tasmania	-43.53448	17.05728	.177	-95.4982	8.4293

Victoria	17.32373	8.45375	.450	-8.4300	43.0775

*. The mean difference is significant at the 0.05 level.

KRUSKALL – WALLIS TESTING OF ST TIME SCHEDULED BY SES TERTILE

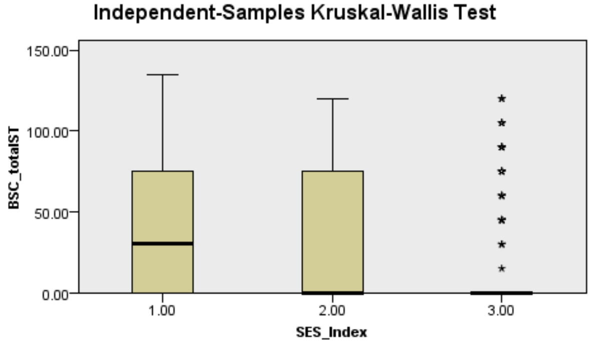

**Total N**	371
**Test Statistic**	24.490
**Degrees of Freedom**	2
**Asymptotic Sig. (2-sided test)**	.000

1. The test statistic is adjusted for ties.

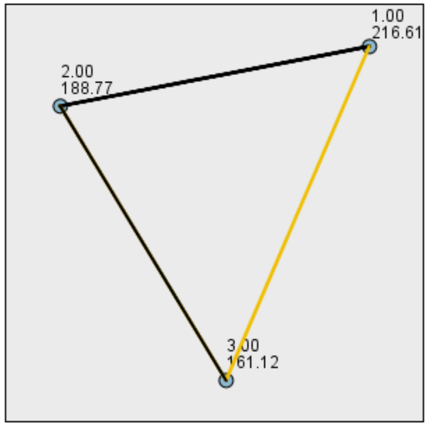

Each node shows the sample average rank of SES_Index.

Sample1-Sample2	Test Statistic	Std. Error	Std. Test Statistic	Sig.	Adj.Sig.
3.00-2.00	27.655	11.914	2.321	.020	.061
3.00-1.00	55.496	11.242	4.937	.000	.000
2.00-1.00	27.841	12.628	2.205	.027	.082

Each row tests the null hypothesis that the Sample 1 and Sample 2 distributions are the same.
Asymptotic significances (2-sided tests) are displayed. The significance level is .05.
Significance values have been adjusted by the Bonferroni correction for [illegible]

BSC ST

Independent-Samples Kruskal-Wallis Test

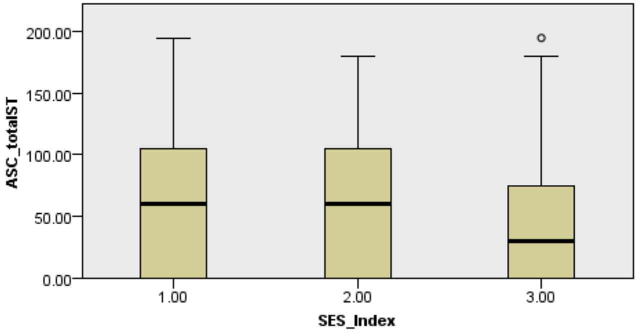

**Total N**	367
**Test Statistic**	6.694
**Degrees of Freedom**	2
**Asymptotic Sig. (2-sided test)**	.035

1. The test statistic is adjusted for ties.

Pairwise Comparisons of SES_Index

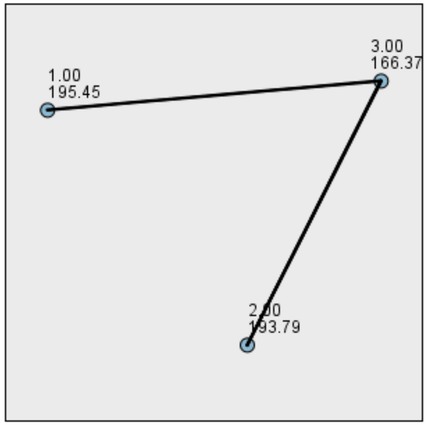

Each node shows the sample average rank of SES_Index.

Sample1-Sample2	Test Statistic	Std. Error	Std. Test Statistic	Sig.	Adj.Sig.
**3.00-2.00**	27.415	13.496	2.031	.042	.127
**3.00-1.00**	29.080	12.422	2.341	.019	.058
**2.00-1.00**	1.666	13.665	.122	.903	1.000

Each row tests the null hypothesis that the Sample 1 and Sample 2 distributions are the same.
Asymptotic significances (2-sided tests) are displayed. The significance level is .05.
Significance values have been adjusted by the Bonferroni correction for [illegible]

ASC ST

KRUSKALL – WALLIS TESTING OF ST TIME SCHEDULED BY STATE

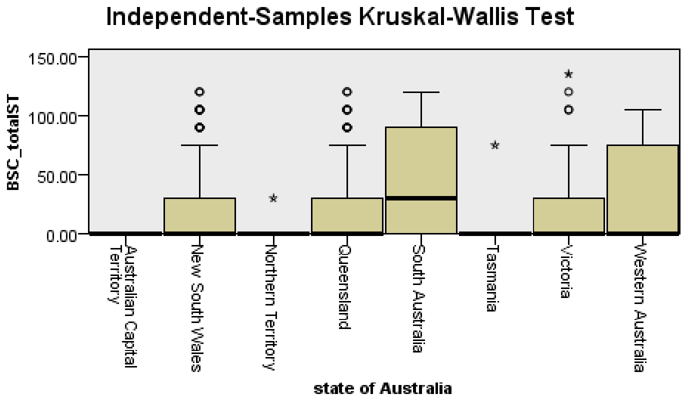

**Total N**	426
**Test Statistic**	30.565
**Degrees of Freedom**	7
**Asymptotic Sig. (2-sided test)**	.000

1. The test statistic is adjusted for ties.

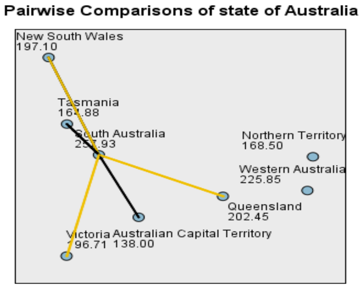

Each node shows the sample average rank of state of Australia.

Sample1-Sample2	Test Statistic	Std. Error	Std. Test Statistic	Sig.	Adj.Sig.
Australian Capital Territory-Tasmania	-26.875	56.813	-.473	.636	1.000
Australian Capital Territory-Northern Territory	-30.500	63.700	-.479	.632	1.000
Australian Capital Territory-Victoria	-58.713	45.009	-1.304	.192	1.000
Australian Capital Territory-New South Wales	-59.100	43.965	-1.344	.179	1.000
Australian Capital Territory-Queensland	-64.453	44.436	-1.450	.147	1.000
Australian Capital Territory-Western Australia	-87.850	47.046	-1.867	.062	1.000
Australian Capital Territory-South Australia	-119.929	44.145	-2.717	.007	.185
Tasmania-Northern Territory	3.625	59.972	.060	.952	1.000
Tasmania-Victoria	-31.838	39.557	-.805	.421	1.000
Tasmania-New South Wales	32.225	38.364	.840	.401	1.000
Tasmania-Queensland	37.578	38.904	.966	.334	1.000
Tasmania-Western Australia	-60.975	41.859	-1.457	.145	1.000
Tasmania-South Australia	93.054	38.571	2.413	.016	.444
Northern Territory-Victoria	-28.213	48.936	-.577	.564	1.000
Northern Territory-New South Wales	28.600	47.977	.596	.551	1.000
Northern Territory-Queensland	-33.953	48.409	-.701	.483	1.000
Northern Territory-Western Australia	-57.350	50.815	-1.129	.259	1.000
Northern Territory-South Australia	-89.429	48.142	-1.858	.063	1.000
Victoria-New South Wales	.387	16.430	.024	.981	1.000
Victoria-Queensland	5.740	17.652	.325	.745	1.000
Victoria-Western Australia	-29.137	23.458	-1.242	.214	1.000
Victoria-South Australia	61.216	16.906	3.621	.000	.008
New South Wales-Queensland	-5.353	14.789	-.362	.717	1.000
New South Wales-Western Australia	-28.750	21.387	-1.344	.179	1.000
New South Wales-South Australia	-60.829	13.890	-4.379	.000	.000
Queensland-Western Australia	-23.397	22.340	-1.047	.295	1.000
Queensland-South Australia	-55.476	15.316	-3.622	.000	.008
Western Australia-South Australia	32.079	21.755	1.475	.140	1.000

Independent-Samples Kruskal-Wallis Test

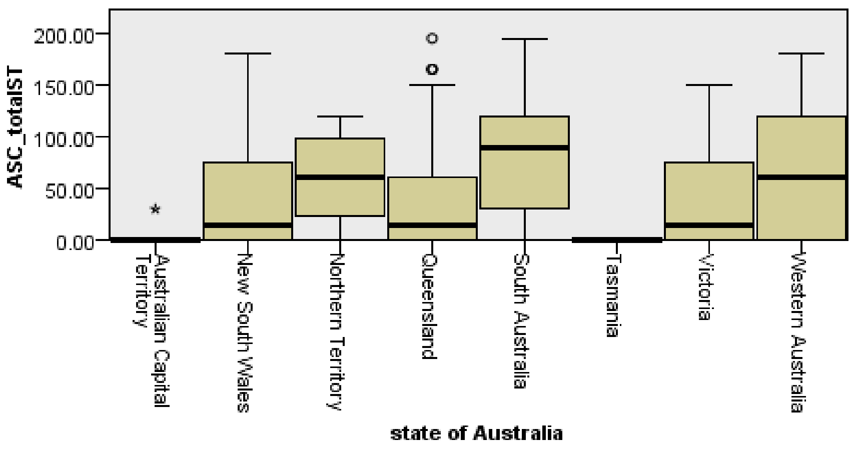

**Total N**	424
**Test Statistic**	48.843
**Degrees of Freedom**	7
**Asymptotic Sig. (2-sided test)**	.000

1. The test statistic is adjusted for ties.

Pairwise Comparisons of state of Australia

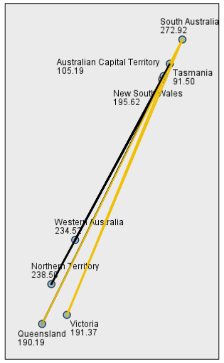

Each node shows the sample average rank of state of Australia.

Sample1-Sample2	Test Statistic	Std. Error	Std. Test Statistic	Sig.	Adj.Sig.
Tasmania-Australian Capital Territory	13.688	63.417	.216	.829	1.000
Tasmania-Queensland	98.689	49.661	1.987	.047	1.000
Tasmania-Victoria	-99.873	50.039	-1.996	.046	1.000
Tasmania-New South Wales	104.121	49.085	2.121	.034	.949
Tasmania-Western Australia	-143.017	52.665	-2.716	.007	.185
Tasmania-Northern Territory	147.000	65.329	2.250	.024	.684
Tasmania-South Australia	181.416	49.342	3.677	.000	.007
Australian Capital Territory-Queensland	-85.002	43.494	-1.954	.051	1.000
Australian Capital Territory-Victoria	-86.186	43.925	-1.962	.050	1.000
Australian Capital Territory-New South Wales	-90.433	42.834	-2.111	.035	.973
Australian Capital Territory-Western Australia	-129.330	46.894	-2.758	.006	.163
Australian Capital Territory-Northern Territory	-133.312	60.773	-2.194	.028	.791
Australian Capital Territory-South Australia	-167.728	43.129	-3.889	.000	.003
Queensland-Victoria	-1.184	19.338	-.061	.951	1.000
Queensland-New South Wales	5.432	16.714	.325	.745	1.000
Queensland-Western Australia	-44.328	25.370	-1.747	.081	1.000
Queensland-Northern Territory	48.311	46.238	1.045	.296	1.000
Queensland-South Australia	-82.727	17.455	-4.739	.000	.000
Victoria-New South Wales	4.248	17.804	.239	.811	1.000
Victoria-Western Australia	-43.144	26.101	-1.653	.098	1.000
Victoria-Northern Territory	47.127	46.643	1.010	.312	1.000
Victoria-South Australia	81.543	18.502	4.407	.000	.000
New South Wales-Western Australia	-38.896	24.221	-1.606	.108	1.000
New South Wales-Northern Territory	-42.879	45.618	-.940	.347	1.000
New South Wales-South Australia	-77.295	15.739	-4.911	.000	.000
Western Australia-Northern Territory	3.983	49.450	.081	.936	1.000
Western Australia-South Australia	38.399	24.738	1.552	.121	1.000
Northern Territory-South Australia	-34.416	45.895	-.750	.453	1.000

Each row tests the null hypothesis that the Sample 1 and Sample 2 distributions are the same.
Asymptotic significances (2-sided tests) are displayed. The significance level is .05.
Significance values have been adjusted by the Bonferroni correction for multiple tests.
