# Supplementary figures and images for: Physical activity and screen time in outside school hours care services across Australia: current versus best practice
Source: BMC Public Health. 2022 Apr 7;22:680. doi: 10.1186/s12889-022-13135-7 (PMC8991463; doi:10.1186/s12889-022-13135-7)

## Supplementary File 4: Feedback on draft guidelines

Feedback on draft guidelines

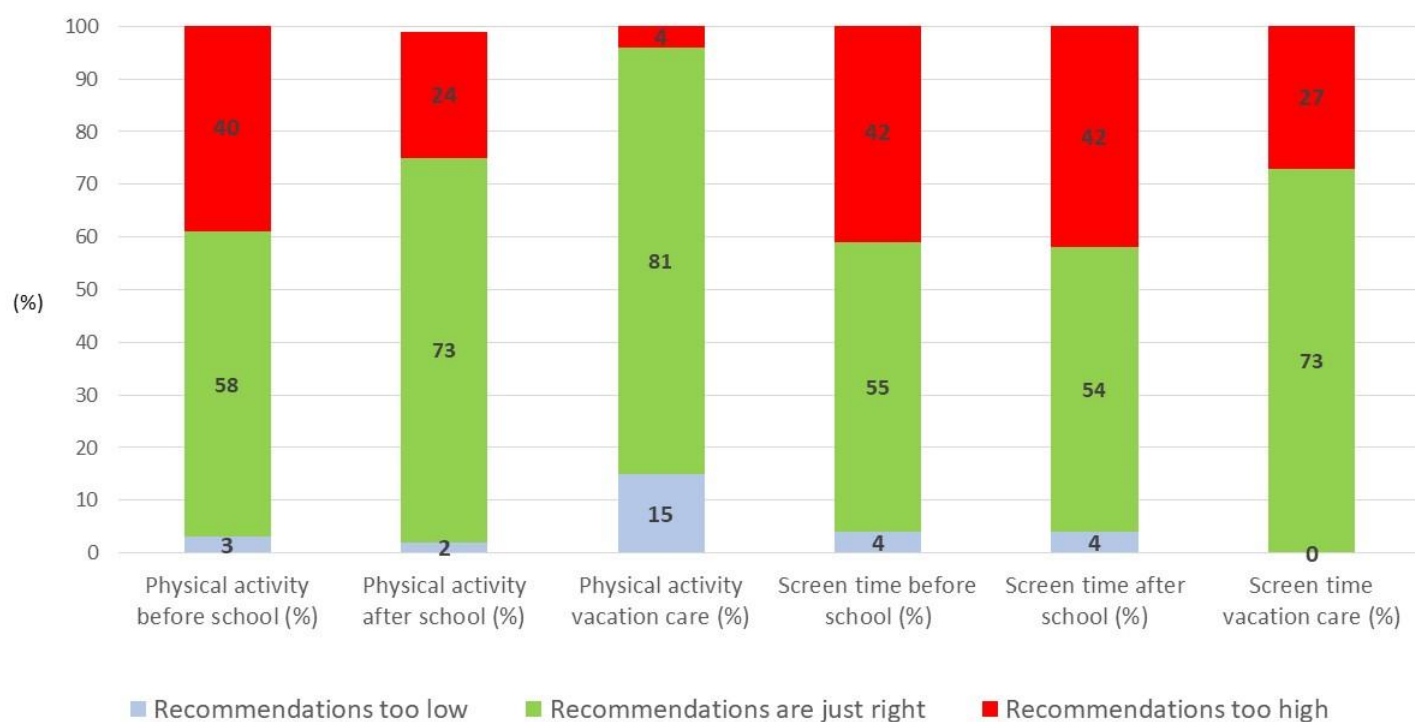

Supplement: Supplementary file 4 — Additional file 4. [file 12889_2022_13135_MOESM4_ESM.pdf]
