# Supplementary material for: Physical activity and screen time in outside school hours care services across Australia: current versus best practice
Source: BMC Public Health. 2022 Apr 7;22:680. doi: 10.1186/s12889-022-13135-7 (PMC8991463; doi:10.1186/s12889-022-13135-7)
Supplement: Supplementary file 5 — Additional file 5. [file 12889_2022_13135_MOESM5_ESM.pdf]

Supplementary File 5: Post Hoc testing meeting guidelines

ANOVA by STATE FOR MEETING INDIVIDUAL GUIDELINES and TOTAL COMPLIANCE

ANOVA

		Sum of Squares	df	Mean Square	F	Sig.
compliance with BSC ST guideline	Between Groups	4.492	7	.642	3.284	.002
	Within Groups	69.956	358	.195		
	Total	74.448	365			
compliance with BSC PA guideline	Between Groups	2.227	7	.318	1.984	.056
	Within Groups	57.404	358	.160		
	Total	59.631	365			
compliance with ASC ST guideline	Between Groups	8.119	7	1.160	5.301	.000
	Within Groups	78.330	358	.219		
	Total	86.448	365			
compliance with ASC PA guideline	Between Groups	.205	7	.029	.372	.918
	Within Groups	28.169	358	.079		
	Total	28.374	365			
number of guidelines met	Between Groups	33.859	7	4.837	6.067	.000
	Within Groups	285.441	358	.797		
	Total	319.301	365			

Multiple Comparisons

Tukey HSD

Dependent Variable	(I) state of Australia	(J) state of Australia	Mean Difference (I-J)	Std. Error	Sig.	95% Confidence Interval Lower Bound	Upper Bound
compliance with BSC ST guideline	Australian Capital Territory	New South Wales	.240	.202	.935	-.38	.86
		Northern Territory	.000	.280	1.000	-.85	.85
		Queensland	.227	.204	.954	-.40	.85
		South Australia	.442	.203	.367	-.18	1.06
		Tasmania	.000	.268	1.000	-.82	.82
		Victoria	.216	.207	.968	-.42	.85
		Western Australia	.360	.217	.712	-.30	1.02
	New South Wales	Australian Capital Territory	-.240	.202	.935	-.86	.38
		Northern Territory	-.240	.202	.935	-.86	.38
		Queensland	-.014	.067	1.000	-.22	.19
		South Australia	.202*	.063	.031	.01	.39
		Tasmania	-.240	.186	.900	-.81	.33
		Victoria	-.025	.076	1.000	-.26	.21
		Western Australia	.120	.098	.927	-.18	.42
	Northern Territory	Australian Capital Territory	.000	.280	1.000	-.85	.85
		New South Wales	.240	.202	.935	-.38	.86
		Queensland	.227	.204	.954	-.40	.85
		South Australia	.442	.203	.367	-.18	1.06

		Tasmania	.000	.268	1.000	-.82	.82
		Victoria	.216	.207	.968	-.42	.85
		Western Australia	.360	.217	.712	-.30	1.02
	Queensland	Australian Capital Territory	-.227	.204	.954	-.85	.40
		New South Wales	.014	.067	1.000	-.19	.22
		Northern Territory	-.227	.204	.954	-.85	.40
		South Australia	.215*	.068	.037	.01	.42
		Tasmania	-.227	.188	.929	-.80	.35
		Victoria	-.011	.080	1.000	-.26	.23
		Western Australia	.133	.102	.896	-.18	.44
	South Australia	Australian Capital Territory	-.442	.203	.367	-1.06	.18
		New South Wales	-.202*	.063	.031	-.39	-.01
		Northern Territory	-.442	.203	.367	-1.06	.18
		Queensland	-.215*	.068	.037	-.42	-.01
		Tasmania	-.442	.186	.256	-1.01	.13
		Victoria	-.226	.077	.066	-.46	.01
		Western Australia	-.082	.099	.992	-.39	.22
	Tasmania	Australian Capital Territory	.000	.268	1.000	-.82	.82
		New South Wales	.240	.186	.900	-.33	.81
		Northern Territory	.000	.268	1.000	-.82	.82
		Queensland	.227	.188	.929	-.35	.80
		South Australia	.442	.186	.256	-.13	1.01
		Victoria	.216	.191	.950	-.37	.80

		Western Australia	.360	.201	.626	-.25	.97
	Victoria	Australian Capital Territory	-.216	.207	.968	-.85	.42
		New South Wales	.025	.076	1.000	-.21	.26
		Northern Territory	-.216	.207	.968	-.85	.42
		Queensland	.011	.080	1.000	-.23	.26
		South Australia	.226	.077	.066	-.01	.46
		Tasmania	-.216	.191	.950	-.80	.37
		Western Australia	.144	.108	.884	-.18	.47
	Western Australia	Australian Capital Territory	-.360	.217	.712	-1.02	.30
		New South Wales	-.120	.098	.927	-.42	.18
		Northern Territory	-.360	.217	.712	-1.02	.30
		Queensland	-.133	.102	.896	-.44	.18
		South Australia	.082	.099	.992	-.22	.39
		Tasmania	-.360	.201	.626	-.97	.25
		Victoria	-.144	.108	.884	-.47	.18
compliance with BSC PA guideline	Australian Capital Territory	New South Wales	.050	.183	1.000	-.51	.61
		Northern Territory	.400	.253	.762	-.37	1.17
		Queensland	-.080	.185	1.000	-.64	.48
		South Australia	.032	.184	1.000	-.53	.59
		Tasmania	-.200	.242	.992	-.94	.54
		Victoria	-.063	.188	1.000	-.63	.51
		Western Australia	.080	.196	1.000	-.52	.68
	New South Wales	Australian Capital Territory	-.050	.183	1.000	-.61	.51

		Northern Territory	.350	.183	.546	-.21	.91
		Queensland	-.130	.061	.389	-.31	.05
		South Australia	-.018	.057	1.000	-.19	.15
		Tasmania	-.250	.168	.814	-.76	.26
		Victoria	-.113	.068	.721	-.32	.10
		Western Australia	.030	.089	1.000	-.24	.30
	Northern Territory	Australian Capital Territory	-.400	.253	.762	-1.17	.37
		New South Wales	-.350	.183	.546	-.91	.21
		Queensland	-.480	.185	.161	-1.04	.08
		South Australia	-.368	.184	.480	-.93	.19
		Tasmania	-.600	.242	.210	-1.34	.14
		Victoria	-.463	.188	.214	-1.03	.11
		Western Australia	-.320	.196	.731	-.92	.28
	Queensland	Australian Capital Territory	.080	.185	1.000	-.48	.64
		New South Wales	.130	.061	.389	-.05	.31
		Northern Territory	.480	.185	.161	-.08	1.04
		South Australia	.112	.062	.618	-.08	.30
		Tasmania	-.120	.170	.997	-.64	.40
		Victoria	.017	.073	1.000	-.20	.24
		Western Australia	.160	.092	.667	-.12	.44
	South Australia	Australian Capital Territory	-.032	.184	1.000	-.59	.53
		New South Wales	.018	.057	1.000	-.15	.19
		Northern Territory	.368	.184	.480	-.19	.93

		Queensland	-.112	.062	.618	-.30	.08
		Tasmania	-.232	.169	.869	-.75	.28
		Victoria	-.094	.070	.876	-.31	.12
		Western Australia	.048	.090	.999	-.23	.32
	Tasmania	Australian Capital Territory	.200	.242	.992	-.54	.94
		New South Wales	.250	.168	.814	-.26	.76
		Northern Territory	.600	.242	.210	-.14	1.34
		Queensland	.120	.170	.997	-.40	.64
		South Australia	.232	.169	.869	-.28	.75
		Victoria	.137	.173	.993	-.39	.66
		Western Australia	.280	.182	.786	-.28	.84
	Victoria	Australian Capital Territory	.063	.188	1.000	-.51	.63
		New South Wales	.113	.068	.721	-.10	.32
		Northern Territory	.463	.188	.214	-.11	1.03
		Queensland	-.017	.073	1.000	-.24	.20
		South Australia	.094	.070	.876	-.12	.31
		Tasmania	-.137	.173	.993	-.66	.39
		Western Australia	.143	.098	.828	-.16	.44
	Western Australia	Australian Capital Territory	-.080	.196	1.000	-.68	.52
		New South Wales	-.030	.089	1.000	-.30	.24
		Northern Territory	.320	.196	.731	-.28	.92
		Queensland	-.160	.092	.667	-.44	.12
		South Australia	-.048	.090	.999	-.32	.23

		Tasmania	-.280	.182	.786	-.84	.28
		Victoria	-.143	.098	.828	-.44	.16
compliance with ASC ST guideline	Australian Capital Territory	New South Wales	.327	.214	.793	-.33	.98
		Northern Territory	.200	.296	.998	-.70	1.10
		Queensland	.240	.216	.954	-.42	.90
		South Australia	.579	.215	.127	-.08	1.23
		Tasmania	.000	.283	1.000	-.86	.86
		Victoria	.353	.219	.744	-.32	1.02
		Western Australia	.560	.229	.224	-.14	1.26
	New South Wales	Australian Capital Territory	-.327	.214	.793	-.98	.33
		Northern Territory	-.127	.214	.999	-.78	.53
		Queensland	-.087	.071	.924	-.30	.13
		South Australia	.252*	.066	.004	.05	.45
		Tasmania	-.327	.196	.710	-.93	.27
		Victoria	.026	.080	1.000	-.22	.27
		Western Australia	.233	.104	.332	-.08	.55
	Northern Territory	Australian Capital Territory	-.200	.296	.998	-1.10	.70
		New South Wales	.127	.214	.999	-.53	.78
		Queensland	.040	.216	1.000	-.62	.70
		South Australia	.379	.215	.644	-.28	1.03
		Tasmania	-.200	.283	.997	-1.06	.66
		Victoria	.153	.219	.997	-.52	.82
		Western Australia	.360	.229	.767	-.34	1.06

	Queensland	Australian Capital Territory	-.240	.216	.954	-.90	.42
		New South Wales	.087	.071	.924	-.13	.30
		Northern Territory	-.040	.216	1.000	-.70	.62
		South Australia	.339*	.072	.000	.12	.56
		Tasmania	-.240	.198	.929	-.85	.37
		Victoria	.113	.085	.887	-.15	.37
		Western Australia	.320	.108	.064	-.01	.65
	South Australia	Australian Capital Territory	-.579	.215	.127	-1.23	.08
		New South Wales	-.252*	.066	.004	-.45	-.05
		Northern Territory	-.379	.215	.644	-1.03	.28
		Queensland	-.339*	.072	.000	-.56	-.12
		Tasmania	-.579	.197	.068	-1.18	.02
		Victoria	-.226	.081	.102	-.47	.02
		Western Australia	-.019	.105	1.000	-.34	.30
	Tasmania	Australian Capital Territory	.000	.283	1.000	-.86	.86
		New South Wales	.327	.196	.710	-.27	.93
		Northern Territory	.200	.283	.997	-.66	1.06
		Queensland	.240	.198	.929	-.37	.85
		South Australia	.579	.197	.068	-.02	1.18
		Victoria	.353	.202	.655	-.26	.97
		Western Australia	.560	.213	.147	-.09	1.21
	Victoria	Australian Capital Territory	-.353	.219	.744	-1.02	.32

		New South Wales	-.026	.080	1.000	-.27	.22
		Northern Territory	-.153	.219	.997	-.82	.52
		Queensland	-.113	.085	.887	-.37	.15
		South Australia	.226	.081	.102	-.02	.47
		Tasmania	-.353	.202	.655	-.97	.26
		Western Australia	.207	.114	.612	-.14	.56
	Western Australia	Australian Capital Territory	-.560	.229	.224	-1.26	.14
		New South Wales	-.233	.104	.332	-.55	.08
		Northern Territory	-.360	.229	.767	-1.06	.34
		Queensland	-.320	.108	.064	-.65	.01
		South Australia	.019	.105	1.000	-.30	.34
		Tasmania	-.560	.213	.147	-1.21	.09
		Victoria	-.207	.114	.612	-.56	.14
compliance with ASC PA guideline	Australian Capital Territory	New South Wales	.096	.128	.995	-.30	.49
		Northern Territory	.200	.177	.951	-.34	.74
		Queensland	.080	.130	.999	-.32	.48
		South Australia	.095	.129	.996	-.30	.49
		Tasmania	.000	.170	1.000	-.52	.52
		Victoria	.059	.131	1.000	-.34	.46
		Western Australia	.080	.137	.999	-.34	.50
	New South Wales	Australian Capital Territory	-.096	.128	.995	-.49	.30
		Northern Territory	.104	.128	.993	-.29	.50
		Queensland	-.016	.042	1.000	-.15	.11
		South Australia	-.001	.040	1.000	-.12	.12

		Tasmania	-.096	.118	.992	-.46	.26
		Victoria	-.037	.048	.994	-.18	.11
		Western Australia	-.016	.062	1.000	-.21	.17
	Northern Territory	Australian Capital Territory	-.200	.177	.951	-.74	.34
		New South Wales	-.104	.128	.993	-.50	.29
		Queensland	-.120	.130	.983	-.52	.28
		South Australia	-.105	.129	.992	-.50	.29
		Tasmania	-.200	.170	.938	-.72	.32
		Victoria	-.141	.131	.962	-.54	.26
		Western Australia	-.120	.137	.988	-.54	.30
	Queensland	Australian Capital Territory	-.080	.130	.999	-.48	.32
		New South Wales	.016	.042	1.000	-.11	.15
		Northern Territory	.120	.130	.983	-.28	.52
		South Australia	.015	.043	1.000	-.12	.15
		Tasmania	-.080	.119	.998	-.44	.28
		Victoria	-.021	.051	1.000	-.18	.13
		Western Australia	.000	.065	1.000	-.20	.20
	South Australia	Australian Capital Territory	-.095	.129	.996	-.49	.30
		New South Wales	.001	.040	1.000	-.12	.12
		Northern Territory	.105	.129	.992	-.29	.50
		Queensland	-.015	.043	1.000	-.15	.12
		Tasmania	-.095	.118	.993	-.45	.27
		Victoria	-.036	.049	.996	-.18	.11

		Western Australia	-.015	.063	1.000	-.21	.18
	Tasmania	Australian Capital Territory	.000	.170	1.000	-.52	.52
		New South Wales	.096	.118	.992	-.26	.46
		Northern Territory	.200	.170	.938	-.32	.72
		Queensland	.080	.119	.998	-.28	.44
		South Australia	.095	.118	.993	-.27	.45
		Victoria	.059	.121	1.000	-.31	.43
		Western Australia	.080	.128	.998	-.31	.47
	Victoria	Australian Capital Territory	-.059	.131	1.000	-.46	.34
		New South Wales	.037	.048	.994	-.11	.18
		Northern Territory	.141	.131	.962	-.26	.54
		Queensland	.021	.051	1.000	-.13	.18
		South Australia	.036	.049	.996	-.11	.18
		Tasmania	-.059	.121	1.000	-.43	.31
		Western Australia	.021	.068	1.000	-.19	.23
	Western Australia	Australian Capital Territory	-.080	.137	.999	-.50	.34
		New South Wales	.016	.062	1.000	-.17	.21
		Northern Territory	.120	.137	.988	-.30	.54
		Queensland	.000	.065	1.000	-.20	.20
		South Australia	.015	.063	1.000	-.18	.21
		Tasmania	-.080	.128	.998	-.47	.31
		Victoria	-.021	.068	1.000	-.23	.19
		New South Wales	.713	.409	.657	-.53	1.96

number of guidelines met	Australian Capital Territory	Northern Territory	.800	.565	.849	-.92	2.52
		Queensland	.467	.412	.950	-.79	1.72
		South Australia	1.147	.410	.098	-.10	2.40
		Tasmania	-.200	.541	1.000	-1.85	1.45
		Victoria	.565	.418	.879	-.71	1.84
		Western Australia	1.080	.437	.212	-.25	2.41
	New South Wales	Australian Capital Territory	-.713	.409	.657	-1.96	.53
		Northern Territory	.087	.409	1.000	-1.16	1.33
		Queensland	-.247	.135	.604	-.66	.17
		South Australia	.434*	.127	.016	.05	.82
		Tasmania	-.913	.375	.227	-2.06	.23
		Victoria	-.149	.153	.978	-.61	.32
		Western Australia	.367	.199	.591	-.24	.97
	Northern Territory	Australian Capital Territory	-.800	.565	.849	-2.52	.92
		New South Wales	-.087	.409	1.000	-1.33	1.16
		Queensland	-.333	.412	.993	-1.59	.92
		South Australia	.347	.410	.990	-.90	1.60
		Tasmania	-1.000	.541	.587	-2.65	.65
		Victoria	-.235	.418	.999	-1.51	1.04
		Western Australia	.280	.437	.998	-1.05	1.61
	Queensland	Australian Capital Territory	-.467	.412	.950	-1.72	.79
		New South Wales	.247	.135	.604	-.17	.66
		Northern Territory	.333	.412	.993	-.92	1.59

		South Australia	.681*	.138	.000	.26	1.10
		Tasmania	-.667	.379	.648	-1.82	.49
		Victoria	.098	.162	.999	-.40	.59
		Western Australia	.613	.206	.062	-.02	1.24
	South Australia	Australian Capital Territory	-1.147	.410	.098	-2.40	.10
		New South Wales	-.434*	.127	.016	-.82	-.05
		Northern Territory	-.347	.410	.990	-1.60	.90
		Queensland	-.681*	.138	.000	-1.10	-.26
		Tasmania	-1.347*	.376	.009	-2.49	-.20
		Victoria	-.583*	.155	.005	-1.06	-.11
		Western Australia	-.067	.201	1.000	-.68	.54
	Tasmania	Australian Capital Territory	.200	.541	1.000	-1.45	1.85
		New South Wales	.913	.375	.227	-.23	2.06
		Northern Territory	1.000	.541	.587	-.65	2.65
		Queensland	.667	.379	.648	-.49	1.82
		South Australia	1.347*	.376	.009	.20	2.49
		Victoria	.765	.385	.494	-.41	1.94
		Western Australia	1.280*	.406	.037	.04	2.52
	Victoria	Australian Capital Territory	-.565	.418	.879	-1.84	.71
		New South Wales	.149	.153	.978	-.32	.61
		Northern Territory	.235	.418	.999	-1.04	1.51
		Queensland	-.098	.162	.999	-.59	.40
		South Australia	.583*	.155	.005	.11	1.06

		Tasmania	-.765	.385	.494	-1.94	.41
		Western Australia	.515	.218	.263	-.15	1.18
	Western Australia	Australian Capital Territory	-1.080	.437	.212	-2.41	.25
		New South Wales	-.367	.199	.591	-.97	.24
		Northern Territory	-.280	.437	.998	-1.61	1.05
		Queensland	-.613	.206	.062	-1.24	.02
		South Australia	.067	.201	1.000	-.54	.68
		Tasmania	-1.280*	.406	.037	-2.52	-.04
		Victoria	-.515	.218	.263	-1.18	.15

*. The mean difference is significant at the 0.05 level.

ANOVA meeting Guidelines by SES

ANOVA

		Sum of Squares	df	Mean Square	F	Sig.
compliance with BSC ST guideline	Between Groups	3.717	2	1.858	9.206	.000
	Within Groups	63.788	316	.202		
	Total	67.505	318			
compliance with BSC PA guideline	Between Groups	.268	2	.134	.909	.404
	Within Groups	46.547	316	.147		
	Total	46.815	318			
compliance with ASC ST guideline	Between Groups	1.400	2	.700	2.956	.053
	Within Groups	74.832	316	.237		
	Total	76.232	318			
compliance with ASC PA guideline	Between Groups	.146	2	.073	1.007	.366
	Within Groups	22.895	316	.072		
	Total	23.041	318			
number of guidelines met	Between Groups	14.879	2	7.440	8.888	.000
	Within Groups	264.506	316	.837		
	Total	279.386	318			

Multiple Comparisons

Tukey HSD

Dependent Variable	(I) SES_Index	(J) SES_Index	Mean Difference (I-J)	Std. Error	Sig.	95% Confidence Interval Lower Bound	95% Confidence Interval Upper Bound
compliance with BSC ST guideline	1.00	2.00	-.144	.066	.073	-.30	.01
		3.00	-.254*	.059	.000	-.39	-.11
	2.00	1.00	.144	.066	.073	-.01	.30
		3.00	-.110	.062	.184	-.26	.04
	3.00	1.00	.254*	.059	.000	.11	.39
		2.00	.110	.062	.184	-.04	.26
compliance with BSC PA guideline	1.00	2.00	-.028	.056	.875	-.16	.10
		3.00	-.067	.051	.378	-.19	.05
	2.00	1.00	.028	.056	.875	-.10	.16
		3.00	-.040	.053	.735	-.17	.09
	3.00	1.00	.067	.051	.378	-.05	.19
		2.00	.040	.053	.735	-.09	.17
compliance with ASC ST guideline	1.00	2.00	-.014	.071	.977	-.18	.15
		3.00	-.141	.064	.074	-.29	.01
	2.00	1.00	.014	.071	.977	-.15	.18
		3.00	-.126	.068	.149	-.29	.03
	3.00	1.00	.141	.064	.074	-.01	.29
		2.00	.126	.068	.149	-.03	.29
compliance with ASC PA guideline	1.00	2.00	.017	.039	.899	-.08	.11
		3.00	-.034	.036	.613	-.12	.05

	2.00	1.00	-.017	.039	.899	-.11	.08
		3.00	-.051	.037	.364	-.14	.04
	3.00	1.00	.034	.036	.613	-.05	.12
		2.00	.051	.037	.364	-.04	.14
number of guidelines met	1.00	2.00	-.169	.134	.416	-.48	.15
		3.00	-.496*	.121	.000	-.78	-.21
	2.00	1.00	.169	.134	.416	-.15	.48
		3.00	-.327*	.127	.028	-.63	-.03
	3.00	1.00	.496*	.121	.000	.21	.78
		2.00	.327*	.127	.028	.03	.63

*. The mean difference is significant at the 0.05 level.
